# The Bulk Breast Cancer Cell and Breast Cancer Stem Cell Activity of Binuclear Copper(II)‐Phenanthroline Complexes

**DOI:** 10.1002/chem.202301188

**Published:** 2023-07-13

**Authors:** Priscilla B. Osei, Joshua Northcote‐Smith, Jiaxin Fang, Kuldip Singh, Fabrizio Ortu, Kogularamanan Suntharalingam

**Affiliations:** ^1^ School of Chemistry University of Leicester Leicester UK

**Keywords:** copper, cancer stem cell, bioinorganic chemistry, antitumour agent, artificial metallonuclease

## Abstract

Mononuclear copper(II)‐phenanthroline complexes have been widely investigated as anticancer agents whereas multinuclear copper(II)‐phenanthroline complexes are underexplored. Here the synthesis and characterisation of two new binuclear copper(II)‐phenanthroline complexes **1** and **2** is reported, comprising of 2,9‐dimethyl‐1,10‐phenanthroline or 2,9‐dimethyl‐4,7‐diphenyl‐1,10‐phenanthroline, terminal chloride ligands, and bridging chloride or hydroxide ligands. The binuclear copper(II) complex containing 2,9‐dimethyl‐1,10‐phenanthroline **1** displays nanomolar toxicity towards bulk breast cancer cells and breast cancer stem cells (CSCs) grown in monolayers, >50‐fold greater than cisplatin (an anticancer metallodrug) and salinomycin (a gold‐standard anti‐CSC agent). Spectacularly, **1** exhibits >100‐fold greater potency toward three‐dimensionally cultured mammospheres than cisplatin and salinomycin. Mechanistic studies show that **1** evokes breast CSC apoptosis by elevating intracellular reactive oxygen species levels and damaging genomic DNA (possibly by an oxidative mechanism). To the best of our knowledge, this is the first study to probe the anti‐breast CSC properties of binuclear copper(II)‐phenanthroline complexes.

## Graphical abstract

**Investigations of the anti‐cancer stem cell (CSC) properties** of multinuclear metal complexes are rare. Here the synthesis, characterisation, and bulk breast cancer cell and breast CSC activity of two new binuclear copper(II) complexes are reported. The mechanism of action of the most stable and potent binuclear copper(II) complex were elucidated.

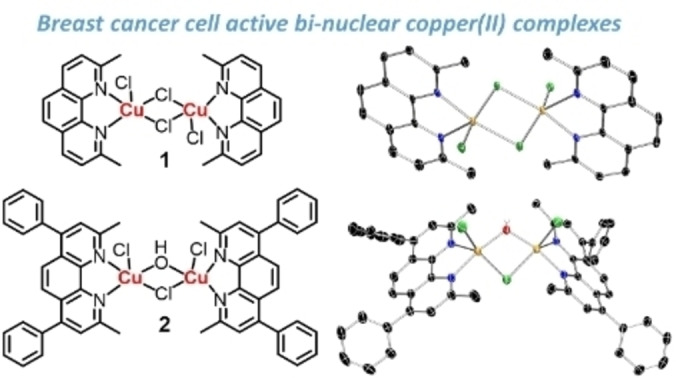

## Introduction

Cancer is a leading cause of death worldwide, with the global burden expected to increase to 28.4 million cases by 2040.^[1]^ Metallopharmaceuticals containing platinum (cisplatin, carboplatin, and oxaliplatin) and arsenic (arsenic trioxide) are a major class of chemotherapeutics used to treat cancer.^[2]^ Although these metallopharmaceuticals are highly effective against certain cancer types, they are unselective which means they suffer from dose‐limiting side effects.^[3]^ These metallopharmaceuticals are also susceptible to inherent or acquired resistance, and are unable to remove a sub‐set of tumour cells called cancer stem cells (CSCs) that are partly responsible for relapse and metastasis.^[3]^ Drawbacks associated with the current crop of clinically approved anticancer metallopharmaceuticals have prompted several investigations into the development of new transition metal complexes as anticancer agents.^[4]^

Copper is an endogenous metal of which an average human body contains about 100 mg.^[5]^ The unique chemical and physical properties of copper have been harnessed to develop bioactive copper‐containing compounds to treat various ailments (e.g. copper deficiency, inflammation, rheumatoid arthritis, and thrombotic disease).^[6]^ Over the last half a century, copper complexes have also been widely studied as potential anticancer agents.^[7]^ The mechanism of action of these copper complexes are diverse, however, a large proportion kill cancer cells by inducing oxidative stress through the generation of reactive oxygen species (ROS).^[7]^ The tendency of copper complexes to elevate ROS levels in cancer cells and thus kill them, is associated to their inherent ability to undergo efficient redox cycling between the copper(I) and copper(II) states under physiological conditions.^[7]^ The cytotoxic mechanism of anticancer copper complexes also include, but are not limited to, topoisomerase I,II inhibition, proteasome disruption, and DNA‐binding and cleavage.[^7^, ^8^] The most clinical advanced anticancer copper complexes, called Casiopeinas, are currently in Phase I clinical trials in Mexico.^[9]^ Casiopeinas comprise of copper(II) bound to bidentate polypyridyl ligands and bidentate *α*‐L‐amino acidato or acetylacetone ligands.^[10]^ A major clinical limitation of the Casiopeinas family of complexes, akin to most anticancer copper(II) complexes developed thus far, is speciation and copper leaching in biological systems and fluids.^[11]^

Copper(II)‐phenanthroline complexes are one of the most widely studied classes of anticancer copper agents.^[12]^ Interest in this class of compounds arose from the serendipitous discovery of the DNA nuclease activity of *bis*(1,10‐phenanthroline)copper(II) in 1979.^[13]^ Mechanistic studies suggest that nuclease activity results from ROS produced from Fenton‐type reactions between the copper(II)‐phenanthroline moiety and molecular oxygen.[^13^, ^14^] Over the last few decades, several copper(II)‐phenanthroline complexes (mono‐ and bis‐phenanthroline and mixed ligand systems) have been reported to effectively cleave DNA, produce ROS, and kill bulk cancer cells.^[12]^ Nevertheless, only a handful of studies have looked into the anti‐CSC properties of copper(II)‐phenanthroline complexes.^[15]^ We have shown that copper(II)‐phenanthroline complexes containing non‐steroidal anti‐inflammatory drugs (NSAIDs) are able to selectively kill breast CSCs over other cell types (within the micro‐ to submicro‐molar concentration range).^[16]^ The mechanism of cytotoxicity of the copper(II)‐phenanthroline complexes, with respect to breast CSCs, partly involves intracellular ROS elevation, activation of oxidative stress pathways, and caspase‐dependent apoptosis.[^16a^, ^16b^] Despite the growing interest in studying the anticancer potential of copper(II)‐phenanthroline complexes, no multinuclear copper(II)‐phenanthroline complexes have been challenged with CSCs of any tissue type.

**Figure 1:**
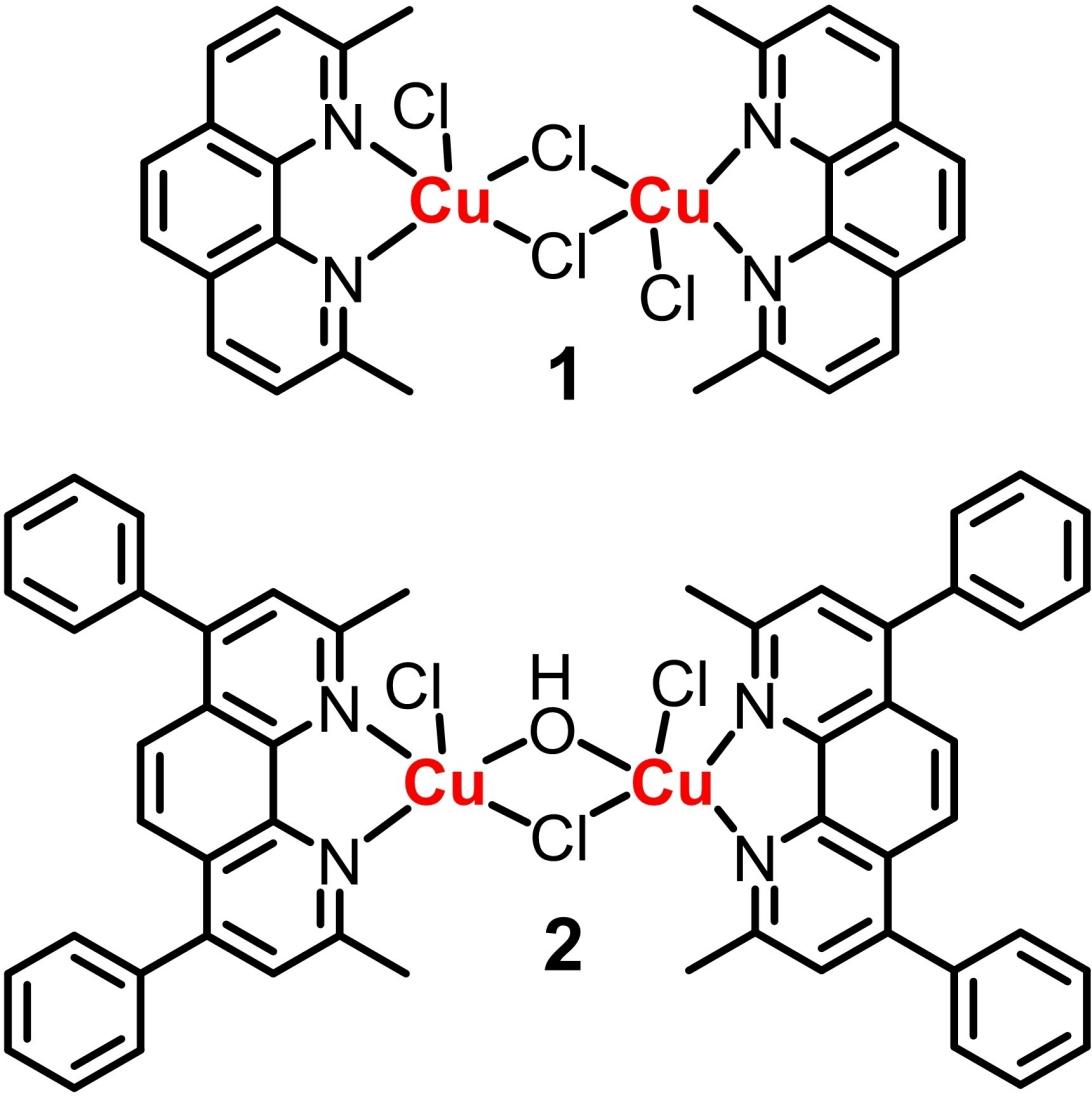
Chemical structures of the binuclear copper(II)‐phenanthroline complexes **1** and **2**.

It is widely accepted that breast CSCs maintain relatively low levels of ROS compared to bulk cancer cells.^[17]^ The low ROS concentration in breast CSCs is associated with increased expression of free radical scavenging systems. This protects breast CSCs from biomolecule damage and contributes to chemo‐ and radio‐resistance. As breast CSCs have adapted to thrive in ROS‐deficient conditions, the intracellular redox state in CSCs is thought to be finely regulated. Therefore, the application of direct or indirect ROS‐inducers represents a potentially efficacious strategy for eliminating breast CSCs. The redox status of bulk breast cancer cells (HMLER) and breast CSCs (HMLER‐shEcad) is well defined in our hands. Herein we explore the bioactivity of binuclear copper(II)‐phenanthroline complexes, with the inherent ability to generate ROS, within bulk breast cancer cell and breast CSC cultures. More specifically, we report the synthesis, characterisation, and anti‐breast cancer activity of two new binuclear copper(II)‐phenanthroline complexes with chloride or hydroxide bridging ligands, **1** and **2** (see Figure 1 for chemical structures). The mechanism of action of the most effective complex **1** in terms of intracellular ROS generation, DNA damage, and apoptosis induction is also reported.

## Results and Discussion

### Synthesis and Characterisation

The binuclear copper(II)‐phenanthroline complexes **1** and **2** investigated in this study are shown in Figure 1. Equimolar amounts of copper(II) chloride and 2,9‐dimethyl‐1,10‐phenanthroline or 2,9‐dimethyl‐4,7‐diphenyl‐1,10‐phenanthroline were reacted in dichloromethane:methanol (1 : 1) to give **1** and **2** in reasonable yields (15–35 %) as a green or red‐brown solid, respectively. The binuclear copper(II) complexes **1** and **2** were fully characterised by high‐resolution TOF mass spectrometry, infrared spectroscopy, and elemental analysis (see Supporting Information). Distinctive molecular ion peaks corresponding to **1** and **2** with the appropriate isotopic pattern were observed in the HR ESI‐TOF MS (*m/z*=648.9620 [**1**‐Cl]^+^; 953.0903 [**2**‐OH]^+^; Figures S1–2). The IR spectra for **1** and **2** displayed C=N and C=C bands in the 1400–1600 cm^−1^ region associated to the phenanthroline‐based ligands (Figure S3). The purity of **1** and **2** was confirmed by elemental analysis (see Supporting Information).

**Figure 2:**
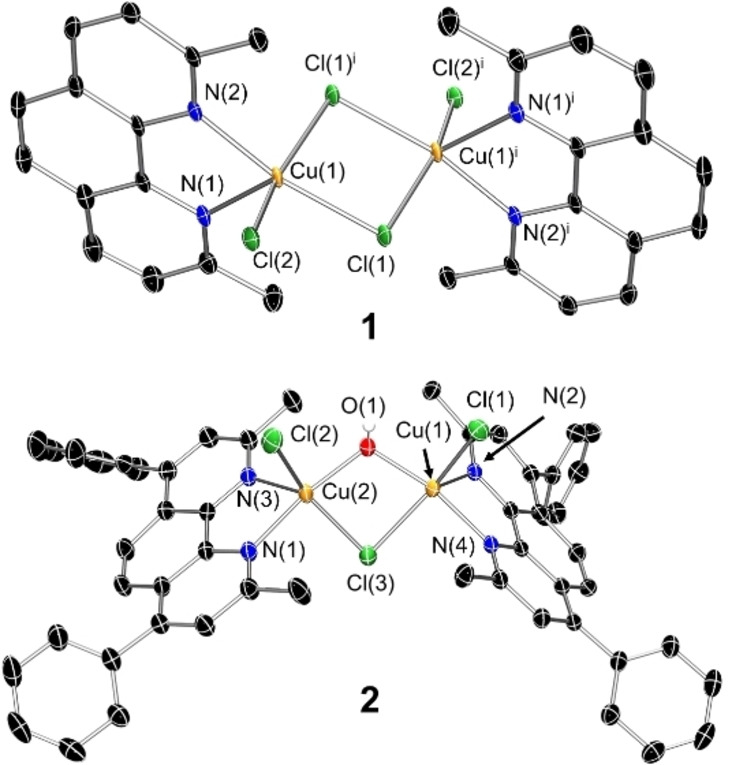
Crystal structures of **1** (top) and **2** (bottom). Ellipsoids are set at 50 % probability level. Hydrogen atoms have been omitted for clarity, with the exceptions of atom H(1) in **2**. Symmetry operation to generate equivalent atoms in **1**: i=1−*x*, −y, 1−*z*.

Single crystals of **1** and **2** suitable for X‐ray diffraction studies were obtained by vapour diffusion of diethyl ether into a methanolic solution of **1** and **2** (CCDC 2250994–2250995, Figure 2, Table S1). Selected bond distances and angles are presented in Tables S2–3. The structure of **1** and **2** both consist of two copper(II) centres with a distorted square pyramidal geometry. Each of the copper(II) centres in **1** are coordinated to 2,9‐dimethyl‐1,10‐phenanthroline, a terminal chloride ligand, and two bridging chloride ligands (Figure 2). Within the NCl_3_ square‐base, the bond angles average to 89.03°, and the bond angles from the apex N(1)/N(1)^i^ atom to the other atoms in the square‐base average to 98.18°. The two Cu atoms reside 0.332 Å away from the NCl_3_ plane. This is consistent with a distorted square pyramidal geometry (*τ*_5_=0.22). Each of the copper(II) centres in **2** are coordinated to 2,9‐dimethyl‐4,7‐diphenyl‐1,10‐phenanthroline, a terminal chloride ligand, a bridging chloride ligand, and a bridging hydroxide ligand (Figure 2). The bridging hydroxide ligand forms significantly shorter bonds to the copper(II) centres than the bridging chloride ligand (1.9148 Å average versus 2.4148 Å average). Within the NOCl_2_ square‐base, the bond angles average to 89.19°, and the bond angles from the apex N(2)/N(3) atom to the other atoms in the square‐base average to 97.85–98.62°. The two Cu atoms reside 0.303–0.330 Å away from the NOCl_2_ plane. This is consistent with a distorted square pyramidal geometry (*τ*_5_=0.19–0.31). Overall, the Cu−N, Cu−Cl, and Cu−O bond lengths observed for **1** and **2** are consistent with bond parameters reported for related binuclear copper(II) complexes.^[18]^

### Lipophilicity and Solution Stability

The lipophilicity of the binuclear copper(II) complexes **1** and **2** was determined by measuring the extent to which it partitioned between octanol and water, P. The experimentally determined LogP values for **1** and **2** was −0.74±0.02 and −0.20±0.02, respectively. The amphiphilic nature of **1** and **2** implies that the binuclear copper(II) complexes should be readily solubilised in aqueous solutions used for cell studies and be readily internalised by dividing cells. UV‐Vis spectroscopy and mass spectrometry studies were carried out to assess the stability of **1** and **2** in biological relevant solutions. In H_2_O : DMSO (200 : 1) and PBS : DMSO (200 : 1), the absorbance bands associated to **1** (50 μM) remained unaltered over the course of 24 h at 37 °C, indicative of good stability (Figures S4‐S5). In contrast, the absorbance of the π‐π* and MLCT bands associated to **2** (50 μM) decreased by 71 % in H_2_O : DMSO (200 : 1) and PBS : DMSO (200 : 1) over the same timeframe (Figures S6‐S7). Nevertheless, the wavelengths associated to the π‐π* and MLCT bands for **2** remained unaltered. Overall, this suggests that **2** is only marginally stable under these conditions.

**Figure 3:**
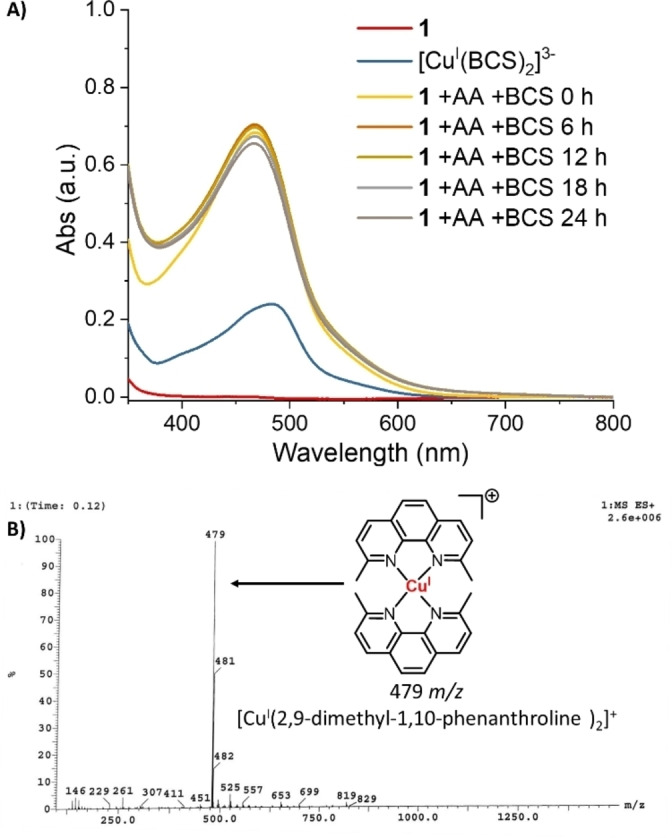
(A) UV‐Vis spectrum of **1** (50 μM) in the presence of ascorbic acid (500 μM) and bathocuproine disulfonate, BCS (100 μM) in PBS : DMSO (200 : 1) over the course of 24 h at 37 °C. (B) ESI mass spectrum (positive mode) of **1** (500 μM) in H_2_O : DMSO (10 : 1) in the presence of ascorbic acid (5 mM) after 24 h incubation at 37 °C.

In PBS : DMSO (200 : 1) with ascorbic acid, glutathione or NADH (10 equivalents), well‐known cellular reductants, the absorbance of **1** and **2** (25 or 50 μM) changed dramatically over the course of 24 h at 37 °C (Figures S8‐S13), implying that **1** and **2** had undergone structural modifications under these conditions. In the presence of bathocuproine disulfonate (BCS, 2 equivalents), a strong copper(I) chelator, and ascorbic acid (10 equivalents) in PBS : DMSO (200 : 1), the UV‐Vis traces for **1** and **2** (50 μM) both displayed a distinctive absorbance band at 480 nm corresponding to [Cu^I^(BCS)_2_]^3−^ (Figures 3A and S14). This suggests that the copper centres in **1** and **2** are likely to undergo reduction to the copper(I) form in the presence of ascorbic acid.^[19]^ The ESI (positive) mass spectra of **1** and **2** (500 μM) in the presence of ascorbic acid, glutathione or NADH (10 equivalents), in H_2_O : DMSO (10 : 1) displayed a distinctive molecular ion peak corresponding to [Cu^I^(2,9‐dimethyl‐1,10‐phenanthroline)_2_]^+^ (479 *m/z*) or [Cu^I^(2,9‐dimethyl‐4,7‐diphenyl‐1,10‐phenanthroline)_2_]^+^ (783 *m/z*), respectively, with the appropriate isotopic pattern (Figures 3B and S15‐S19). This suggests that the copper(II) centres in **1** and **2** undergo reduction to copper(I) by cellular reductants triggering cleavage of the bridging chloride or hydroxide bonds and subsequent ligand exchange. Although the identified reduced products of **1** and **2** were [Cu^I^(2,9‐dimethyl‐1,10‐phenanthroline)_2_]^+^ and [Cu^I^(2,9‐dimethyl‐4,7‐diphenyl‐1,10‐phenanthroline)_2_]^+^ they are unlikely to be the primary reduced products formed inside cells. Biomolecules with high affinities for copper(I) ions (containing free nitrogen and sulphur donor atoms) are likely to sequester the reduced copper(I) form of **1** and **2**.

### Cytotoxicity Studies in Monolayer Cell Cultures

The potency of the binuclear copper(II) complexes **1** and **2** towards bulk breast cancer cells (HMLER) and breast CSCs (HMLER‐shEcad) (grown in two‐dimensional cultures) was determined using the well‐established colorimetric MTT (3‐(4,5‐dimethylthiazol‐2‐yl)‐2,5‐diphenyltetrazolium bromide) assay. IC_50_ values (concentration at which cell viability is reduced by 50 %) were determined from dose‐response curves (Figures S20–S21) and are presented in Table 1. The binuclear copper(II) complex **1** displayed nanomolar toxicity towards HMLER and HMLER‐shEcad cells whereas **2** displayed sub‐micromolar toxicity. The greater bulk breast cancer cell and breast CSC potency of **1** over **2** could be related to the greater stability of **1** over **2** in biologically relevant solutions (Figures S4–S7). Notably, **1** and **2** displayed up to 53‐fold and 71‐fold greater potency towards HMLER‐shEcad cells than salinomycin (a well‐established anti‐breast CSC agent) and cisplatin (a clinically used platinum‐based anticancer agent), respectively (Table 1).[^16a^, ^20^] Furthermore, the most active binuclear copper(II) complex **1** is significantly more potent towards breast CSCs than previously reported, structurally related anti‐breast CSC metal complexes: 28‐fold more active than copper(II) complexes containing phenanthroline‐based and indomethacin ligands,^[16a]^ 101‐fold more active than a metallopeptide consisting of Cu(1,10‐phenanthroline)Cl_2_ and a mitochondria‐penetrating peptide,^[21]^ and 105‐fold more active than a tetranuclear copper(II) complexes containing multiple diclofenac and Schiff base moieties.^[22]^

**Table 1:** IC_50_ values of the binuclear copper(II) complexes **1** and **2**, cisplatin, and salinomycin against HMLER cells, HMLER‐shEcad cells, and HMLER‐shEcad mammospheres.

Compound	HMLER IC_50_ [μM]^[a]^	HMLER‐shEcad IC_50_ [μM]^[a]^	Mammosphere IC_50_ [μM]^[b]^
**1**	0.03±0.001	0.08±0.004	0.11±0.001
**2**	0.34±0.02	0.86±0.01	1.87±0.01
salinomycin^[c]^	11.43±0.42	4.23±0.35	18.50±1.50
cisplatin^[c]^	2.57±0.02	5.65±0.30	13.50±2.34

[a] Determined after 72 h incubation (mean of three independent experiments±SD). [b] Determined after 5 days incubation (mean of three independent experiments±SD). [c] Reported in references 16a, 20, 22, and 25.

To gauge therapeutic potential, the cytotoxicity of **1** and **2** towards non‐cancerous epithelial breast MCF10 A cells was determined. The binuclear copper(II) complexes **1** (IC_50_ value=0.09±0.003 μM) and **2** (IC_50_ value=1.01±0.03 μM) were significantly (*p* <0.05, n=18) less potent towards MCF10 A cells than HMLER cells and similarly potent towards HMLER‐shEcad cells (Figure S22). Therefore, according to the cytotoxicity studies in monolayer cultures, **1** and **2** have the potential to preferentially kill bulk breast cancer cells over non‐cancerous breast cells at certain nanomolar or sub‐micromolar concentrations. Control cell viability studies showed that the mononuclear copper(II)‐phenanthroline complexes, Cu(1,10‐phenanthroline)Cl_2_, and Cu(4,7‐diphenyl‐1,10‐phenanthroline)Cl_2_ were significantly (*p*<0.05, n=18) less potent towards bulk breast cancer cells and breast CSCs than the binuclear copper(II) complex **1** (Figures S23–S24, Table S4). This shows that **1** has greater anti‐breast cancer cell activity than its mononuclear analogues.

The ability of **1** and **2** to affect the cell viability of osteosarcoma cells (U2OS) and osteosarcoma stem cells (OSC, U2OS‐MTX) was also determined (Figures S25–S26, Table S5). The binuclear copper(II) complex **1** displayed nanomolar toxicity towards U2OS and U2OS‐MTX cells whereas **2** displayed sub‐micromolar toxicity. The potency of **1** and **2** towards the osteosarcoma cell lines (U2OS and U2OS‐MTX) was similar to their potency towards the breast cancer cell lines (HMLER and HMLER‐shEcad), suggesting that **1** and **2** have a broad activity range.

### Mammosphere Inhibition and Viability Studies

**Figure 4:**
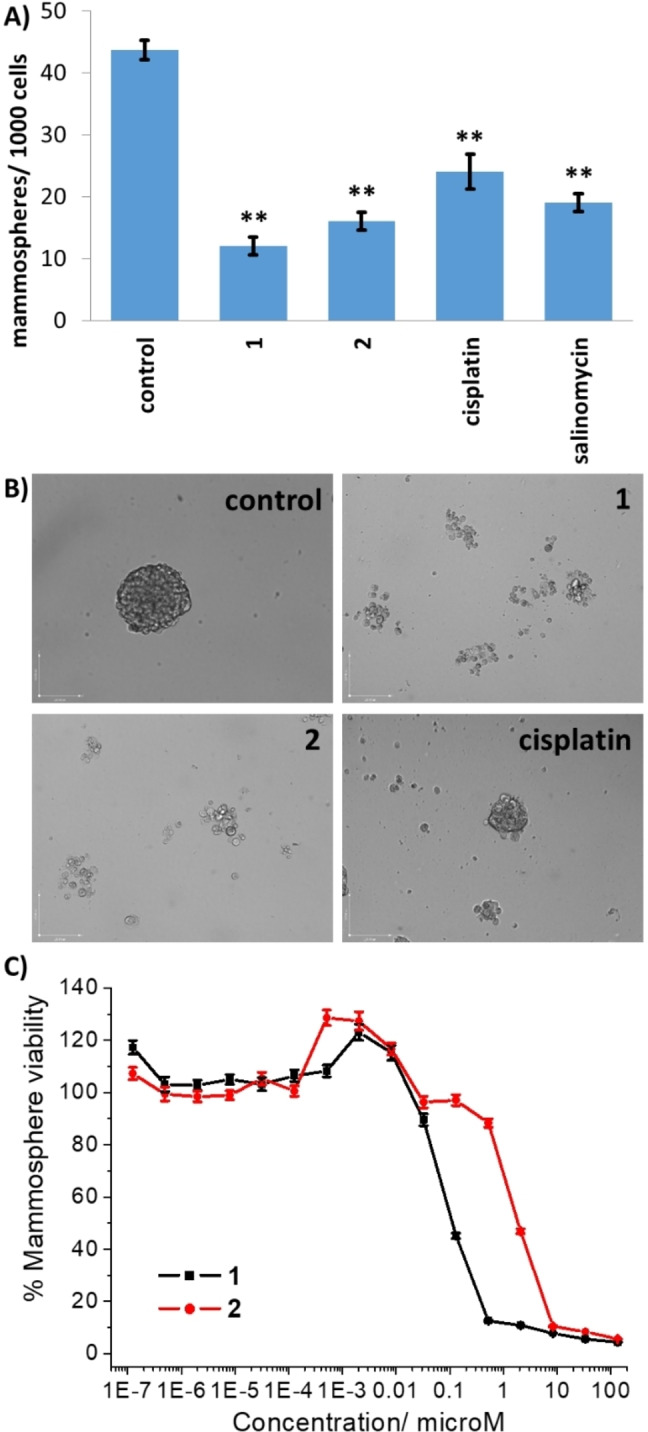
(A) Quantification of mammosphere formation with HMLER‐shEcad cells untreated and treated with **1**, **2**, cisplatin or salinomycin at their respective IC_20_ values for 5 days. Error bars=SD and Student t‐test, **=*p*<0.01. (B) Representative bright‐field images (×10) of the mammospheres in the absence and presence of **1**, **2** or cisplatin at their respective IC_20_ values for 5 days. (C) Representative dose‐response curves for the treatment of HMLER‐shEcad mammospheres with **1** or **2** after 5 days incubation. Error bars=SD.

HMLER‐shEcad cells grown under low attachment, serum‐free conditions can aggregate together to form three‐dimensional spheroids known as mammospheres.^[23]^ Mammospheres are more representative of tumours than two‐dimensional monolayer cell cultures. Furthermore, studies conducted in mammosphere systems provide a good gauge of in vivo and translation potential. Single cell suspensions of HMLER‐shEcad cells were dosed with non‐lethal concentrations (IC_20_ value for 5 days) of binuclear copper(II) complexes **1** and **2** and their ability to inhibit mammosphere formation was measured. Both binuclear copper(II) complexes **1** and **2** completely disrupted mammosphere formation under these conditions with respect to the number and size of mammospheres formed (Figure 4A–B). A slightly reduced mammosphere inhibitory effect was observed for salinomycin and cisplatin (Figures 4A−B and S27). More specifically, **1** and **2** reduced the number of mammospheres formed by 63–73 % compared to the untreated control, whereas salinomycin and cisplatin induced a 45–56 % reduction in the number of mammospheres formed (Figure 4A). To decipher the potency of the binuclear copper(II) complexes **1** and **2** towards HMLER‐shEcad mammospheres, TOX8 a resazurin‐based reagent was used.^[24]^ The IC_50_ values of **1** and **2** (the concentration required to reduce mammosphere viability by 50 %) were interpolated from dose‐response curves (Figure 4C) and are shown in Table 1. The IC_50_ value for **1** was in the sub‐micromolar range whereas the IC_50_ value for **2** was in the low micromolar range. Strikingly the mammosphere potency (based on the IC_50_ values) of **1** was 168‐fold and 123‐fold greater than salinomycin and cisplatin, respectively (Table 1), and 17‐fold higher than **2**.[^22^, ^25^] Overall, the mammosphere studies show that **1** and **2** are able to markedly reduce mammosphere formation, size, and viability, and that the 2,9‐dimethyl‐1,10‐phenanthroline‐bearing complex **1** displayed the greater activity of the two binuclear complexes.

### Mechanism of Action Studies

**Figure 5:**
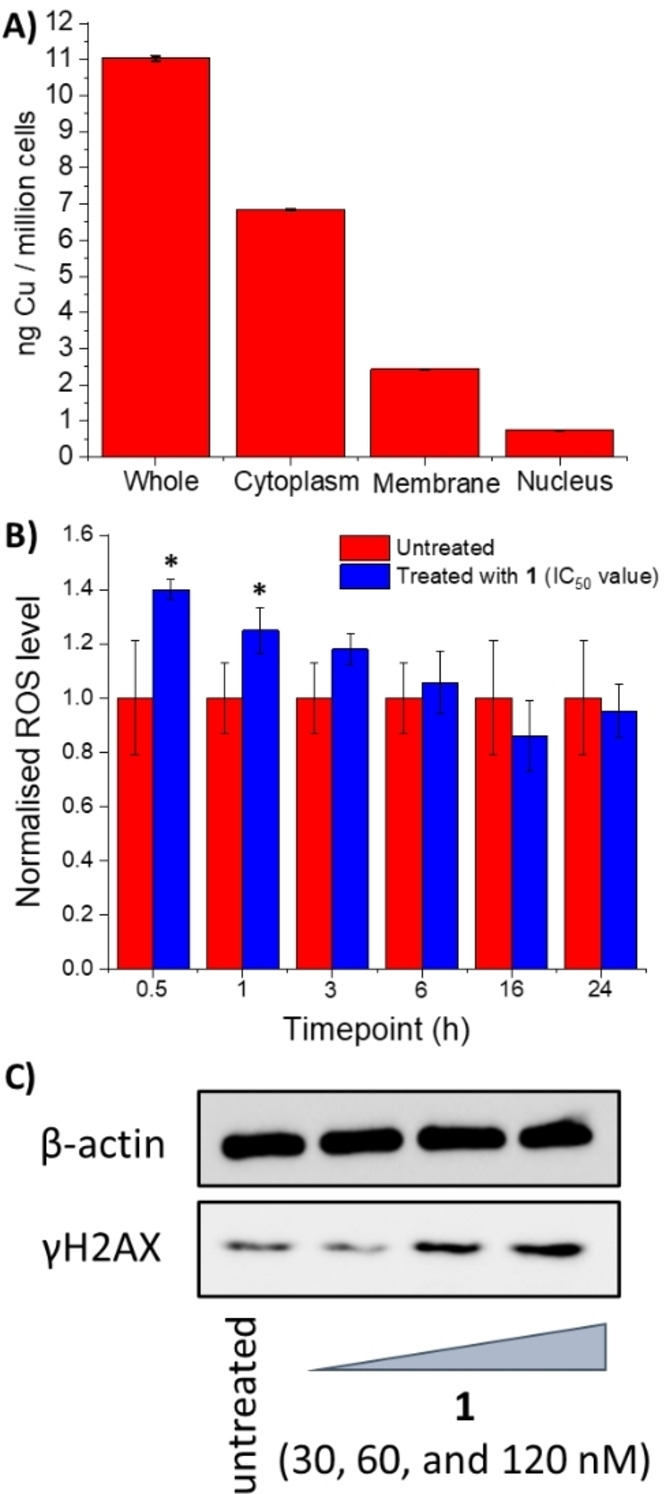
(A) Copper content in whole cell, cytoplasm, nucleus, and membrane fractions isolated from HMLER‐shEcad cells treated with **1** (0.25 μM for 24 h). (B) Normalised ROS activity in untreated HMLER‐shEcad cells (control) and HMLER‐shEcad cells treated with **1** (IC_50_ value ×2, 0.5–24 h). Error bars=SD and Student t‐test, *=*p*<0.05. (C) Immunoblotting analysis of γH2AX related to the DNA damage pathway. Protein expression in HMLER‐shEcad cells following treatment with **1** (0.03–0.12 μM for 24 h).

Given that the 2,9‐dimethyl‐1,10‐phenanthroline‐bearing binuclear complex **1** displayed the greatest solution stability and potency towards breast CSCs cultured in monolayers and three‐dimensional systems, studies were carried out to provide insight into its mechanism of action. First the ability of **1** to enter breast CSCs and its intracellular localisation was determined. HMLER‐shEcad cells were incubated with **1** (0.25 μM for 24 h) and the copper content was determined in the whole cell, cytoplasmic, nuclear, and membrane fractions by inductively coupled plasma mass spectrometry (ICP‐MS). Supplementary dual FITC Annexin V‐propidium iodide flow cytometry studies unambiguously showed that the cell membrane of HMLER‐shEcad cells was not detrimentally affected under these conditions (Figure S28). Given the low administration concentration used (0.25 μM), a reasonable amount of **1** was internalised into breast CSCs (11.03 ng of Cu/ million cells) (Figure 5A). The majority of internalised **1** was detected in the cytoplasm, however, appreciable amounts of **1** were also found in the membrane and nuclear fractions (Figure 5A). The distribution of **1** inside breast CSCs suggests that **1**‐induced breast CSC toxicity could be related to interactions with cytoplasmic biomolecules, however, a genomic DNA‐dependent mechanism could also be possible.

Copper(II)‐phenanthroline complexes have been widely reported to induce cell death by generating intracellular ROS.^[12]^ To determine if the binuclear copper(II) complex **1** elevated intracellular ROS levels in breast CSCs, the ROS levels in HMLER‐shEcad cells was measured upon treatment with **1** (IC_50_ value ×2, 0.5–24 h), using 6‐carboxy‐2’,7’‐dichlorodihydrofluorescein diacetate (DCFH‐DA) (Figure 5B). It should be noted that using DCFH‐DA may not be the best ROS detection method due to possible artefactual amplification of its fluorescence via a redox‐cycling mechanism involving the dichlorodihydrofluorescein radical.^[26]^ Upon incubation of HMLER‐shEcad cells with **1** for 0.5 h a marked increase (40 %) in intracellular ROS levels was observed compared to the untreated control cells (Figure 5B). A steady decrease in ROS levels was observed upon longer exposure times (1–24 h). As the highest elevation in ROS levels was observed at a short timepoint (0.5 h), it is likely that ROS was generated directly by **1**, rather than occurring as a result of the downstream effects of cellular stress or due to cell death.

**Figure 6:**
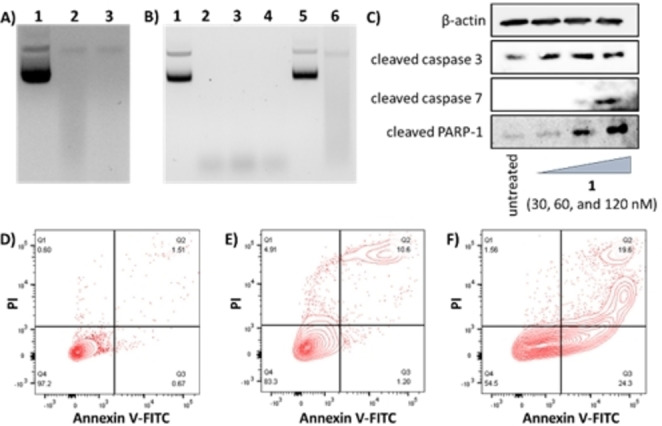
(A) Effect of ascorbic acid on **1**‐mediated DNA cleavage after a 24 h incubation period. Lane 1: DNA only, Lane 2–3: DNA+10 and 20 μM of **1** with 10 equivalents of ascorbic acid. (B) Inhibition of **1**‐mediated DNA cleavage by ^t^BuOH, DMSO, KI, or NaN_3_ after a 24 h incubation period. Lane 1: DNA only, Lane 2: DNA+**1** (10 μM) with 10 equivalents of ascorbic acid, Lane 3–6: DNA+**1** (10 μM) with 10 equivalents of ascorbic acid+^t^BuOH (10 mM), DMSO (10 mM), KI (40 mM) or NaN_3_ (40 mM). (C) Immunoblotting analysis of proteins related to the caspase‐dependent apoptosis pathway. Protein expression in HMLER‐shEcad cells following treatment with **1** (0.03–0.12 μM for 48 h). (D–F) FITC Annexin V‐propidium iodide binding assay plots of untreated HMLER‐shEcad cells, and HMLER‐shEcad cells treated with **1** (IC_50_ value ×2 for 48 h) or cisplatin (25 μM for 48 h).

Given that the binuclear copper(II) complex **1** is able to produce intracellular ROS and have access to the nucleus, immunoblotting studies were carried out to determine if **1** induced DNA damage in CSCs. HMLER‐shEcad cells dosed with **1** (0.03–0.12 μM for 24 h) exhibited a marked increase in the expression of phosphorylated H2AX (γH2AX), indicative of genomic DNA damage (Figure 5C).^[27]^ Copper(II)‐phenanthroline complexes can cleave DNA in a ROS‐dependent fashion.^[14b]^ The DNA nuclease activity of **1** was assessed by agarose gel electrophoresis. Upon incubation of plasmid pUC19 DNA (100 ng) with **1** (10–20 μM for 24 h) in the absence of external reducing agents, no significant cleavage was observed (Figure S29). However, in the presence of ascorbic acid (10 equivalents), **1** induced complete conversion of supercoiled DNA (form I) to several small fragments (at 10–20 μM) (Figure 6A). To determine the oxidative mechanism by which **1** induces DNA cleavage, nuclease activity was probed in the presence of ROS scavengers (^t^BuOH, DMSO, KI and NaN_3_) (Figure 6B). KI and NaN_3_ displayed the greatest inhibitory effect, suggesting that hydrogen peroxide (H_2_O_2_) and singlet oxygen (^1^O_2_) are the major ROS intermediates formed during the DNA cleavage process. To shed light on the DNA‐binding mode of **1** prior to initiating DNA cleavage, the nuclease activity was probed in the presence of a minor groove binder (DAPI, 2‐(4‐amidinophenyl)‐1H‐indole‐6‐carboxamidine, 50 μM), a major groove binder (methyl green, 50 μM) and an intercalator (thiazole orange, 10 μM) (Figure S30). DNA cleavage was inhibited in the presence of all of the DNA binders suggesting that **1** binds to DNA via multiple modes. Collectively, the DNA cleavage, immunoblotting, and intracellular ROS studies suggest that **1** potentially binds to DNA via the grooves and intercalation prior to inducing oxidative DNA cleavage.

DNA damage, when left unrepaired, can lead to apoptosis,^[28]^ therefore the expression of proteins associated to apoptosis were monitored. HMLER‐shEcad cells treated with **1** (0.03–0.12 μM for 48 h) exhibited a clear increase in the level of cleaved caspase 3 and 7, and cleaved PARP‐1 compared to untreated control cells (Figure 6C). This is indicative of caspase‐dependent apoptosis. Apoptosis also induces morphological changes that can lead to cell membrane rearrangement. This process results in the translocation of phosphatidylserine residues to the membrane exterior, which can be detected by Annexin V.^[29]^ Damaged cell membranes also facilitate propidium iodide uptake. Using a dual FITC Annexin V‐propidium iodide staining flow cytometry assay, we explored the occurrence of apoptosis in HMLER‐shEcad cells treated with **1**. Dosage with **1** (IC_50_ value ×2 for 48 h) induced large populations of cells to undergo late‐stage apoptosis (Figure 6D−E). This was comparable to dosage with cisplatin (25 μM for 48 h), a well‐known apoptosis inducer (Figure 6F). Taken together, the immunoblotting and flow cytometry studies show that **1** induces apoptotic CSC death (probably after oxidative DNA cleavage).

## Conclusions

In summary we report the synthesis, characterisation, and anti‐breast CSC properties of two new binuclear copper(II)‐phenanthroline complexes **1** and **2**. X‐ray crystallography studies show that the two copper centres in **1** and **2** adopt distorted square pyramidal geometries (*τ*_5_=0.19–0.31). The copper(II) centres are coordinated to a phenanthroline ligand via two nitrogen atoms (2,9‐dimethyl‐1,10‐phenanthroline for **1** and 2,9‐dimethyl‐4,7‐diphenyl‐1,10‐phenanthroline for **2**), a terminal chloride ligand, and two bridging chloride ligands (for **1**) or one bridging chloride and one bridging hydroxide ligand (for **2**). The 2,9‐dimethyl‐1,10‐phenanthroline‐containing complex **1** was markedly more stable in biologically relevant solutions than the 2,9‐dimethyl‐4,7‐diphenyl‐1,10‐phenanthroline‐containing complex **2**. The binuclear complexes **1** and **2** exhibited significantly better potency towards bulk breast cancer cells and breast CSCs (cultured in monolayers) than cisplatin and salinomycin. The binuclear complexes **1** and **2** were also able to inhibit the formation of mammospheres at sub‐micromolar concentrations with respect to mammospheres size and number. Strikingly, and highly promising from a translational standpoint, the most effective binuclear complex **1** exhibited 168‐ and 123‐fold greater mammosphere potency than salinomycin and cisplatin, respectively. Cell uptake studies indicated that **1** readily enters breast CSCs and is largely localised in the cytoplasm, however appreciable amounts of **1** were also found in the nucleus. Furthermore, **1** can elevate intracellular ROS levels at short exposure times, damage genomic DNA (possibly by oxidative cleavage), and trigger caspase‐dependent apoptosis. As far as we are aware, this is the first report of binuclear copper(II) complexes with promising anti‐breast CSC activities. Our findings reinforce the therapeutic potential of multinuclear metal complexes and more specifically provides the basis for the development of binuclear copper(II)‐phenanthroline complexes as anti‐breast CSC agents.

## Experimental Section

**Materials and methods**: All synthetic procedures were performed under normal atmospheric conditions. Fourier transform infrared (FTIR) spectra were recorded with an IRAffinity‐1S Shimadzu spectrophotometer. Elemental analysis of the compounds prepared was performed commercially by the University of Cambridge. CuCl_2_, 2,9‐dimethyl‐1,10‐phenanthroline, 2,9‐dimethyl‐4,7‐diphenyl‐1,10‐phenanthroline, and Cu(1,10‐phenanthroline)Cl_2_ were purchased from Sigma Aldrich and used as received. Cu(4,7‐diphenyl‐1,10‐phenanthroline)Cl_2_ was prepared according to a previously reported method.^[30]^

**Synthesis of [Cu_2_(2,9‐dimethyl‐1,10‐phenanthroline)_2_Cl_2_(μ‐Cl)_2_] (1)**: 2,9‐Dimethyl‐1,10‐phenanthroline (166.4 mg, 0.8 mmol) dissolved in DCM (1 mL) was added dropwise to CuCl_2_ (107.4 mg, 0.8 mmol) dissolved in MeOH (1 mL). The mixture was stirred at room temperature for 1 h. The resulting precipitate was filtered and washed with H_2_O (3×10 mL) and Et_2_O (3×10 mL) to yield **1** a green solid (84 mg, 15 %); ATR‐FTIR (solid, cm^−1^): 3265, 3052, 1615, 1594, 1563, 1506, 1428, 1366, 1296, 1226, 1146, 1032, 988, 939, 861, 812, 775, 729, 680, 653, 549, 436, 410; HR ESI‐TOF MS Calcd. for C_28_H_24_Cl_3_Cu_2_N_4_ [M−Cl]^+^: 648.9637 a.m.u. Found [M−Cl]^+^: 648.9620 a.m.u.; Anal. Calcd. For **1**, C_28_H_24_Cl_4_Cu_2_N_4_⋅H_2_O (%): C, 47.81; H, 3.73; N, 7.96. Found: C, 47.84; H, 3.84; N, 7.90.

**Synthesis of [Cu_2_(2,9‐dimethyl‐4,7‐diphenyl‐1,10‐phenanthroline)_2_Cl_2_(μ‐Cl)(μ‐OH)] (2)**: 2,9‐Dimethyl‐4,7‐diphenyl‐1,10‐phenanthroline (280.5 mg, 0.8 mmol) dissolved in DCM (1 mL) was added dropwise to CuCl_2_ (107.8 mg, 0.8 mmol) dissolved in MeOH (1 mL). The mixture was stirred at room temperature for 1 h. The resulting precipitate was filtered and washed with H_2_O (3×10 mL) and Et_2_O (3×10 mL) to yield **2** a red‐brown solid (268.7 mg, 35 %); ATR‐FTIR (solid, cm^−1^): 3234, 3042, 1623, 1569, 1549, 1484, 1439, 1400, 1371, 1185, 1078, 1028, 887, 863, 835, 781, 732, 702, 669, 663, 575, 537, 481, 438, 403, 378; HR ESI‐TOF MS Calcd. for C_52_H_40_Cl_3_Cu_2_N_4_ [M−OH]^+^: 953.0894 a.m.u. Found [M‐OH]^+^: 953.0903 a.m.u.; Anal. Calcd. For **1**, C_52_H_41_Cl_3_Cu_2_N_4_O⋅H_2_O (%): C, 63.13; H, 4.38; N, 5.66. Found: C, 63.24; H, 3.95; N, 5.67.

**X‐ray crystallography**: Crystals were mounted in inert oil on glass fibres and transferred to a Bruker D8 Quest diffractometer equipped with a Photon III detector. Intensities were integrated from data recorded on 1° frames by *ω* or *ϕ* rotation. A multiscan method (SADABS) for absorption correction with a beam profile was applied.^[31]^ The structures were solved using SHELXS^[32]^ or SHELXT;^[33]^ the datasets were refined by full‐matrix least‐squares on reflections with *F* ^2^≥2σ(*F* ^2^) values, with anisotropic displacement parameters for all non‐hydrogen atoms, and with constrained riding hydrogen geometries; *U*_iso_(H) was set at 1.2 (1.5 for methyl groups) times *U*_eq_ of the parent atom. The largest features in final difference syntheses were close to heavy atoms and were of no chemical significance. SHELX[^32^, ^33^] was employed through OLEX2^[34]^ for structure solution and refinement. ORTEP‐3^[35]^ and POV‐Ray^[36]^ were employed for molecular graphics.

Deposition Numbers 2250994 (for **2**) and 2250995 (for **1**) contain the supplementary crystallographic data for this paper. These data are provided free of charge by the joint Cambridge Crystallographic Data Centre and Fachinformationszentrum Karlsruhe Access Structures service.

**Measurement of water‐octanol partition coefficient (LogP)**: The LogP values for **1** and **2** were determined using the shake‐flask method and UV‐Vis spectroscopy. The octanol used in this experiment was pre‐saturated with water. An aqueous solution of **1** and **2** (500 μL, 100 μM) was incubated with octanol (500 μL) in a 1.5 mL tube. The tube was shaken at room temperature for 24 h. The two phases were separated by centrifugation and the **1** and **2** content in each phase was determined by UV‐Vis spectroscopy.

**UV‐Vis stability studies**: The UV‐Vis spectra of **1** and **2** (25 or 50 μM) were measured in H_2_O : DMSO (200 : 1) and PBS : DMSO (200 : 1) over the course of 24 h at 37 °C. Similar experiments were also conducted in PBS : DMSO (200 : 1) in the presence of ascorbic acid, glutathione or NADH (10 equivalents), and in the case of ascorbic acid, with and without bathocuproine disulfonate (2 equivalents). The UV‐Vis absorption spectra were recorded on a Cary 3500 UV‐Vis spectrophotometer.

**Cell culture**: The human mammary epithelial cell lines, HMLER and HMLER‐shEcad were kindly donated by Prof. R. A. Weinberg (Whitehead Institute, MIT). The MCF10A human breast epithelial cell line was acquired from American Type Culture Collection (ATCC, Manassas, VA, USA). HMLER, HMLER‐shEcad, and MCF10 A cells were maintained in Mammary Epithelial Cell Growth Medium (MEGM) with supplements and growth factors (BPE, hydrocortisone, hEGF, insulin, and gentamicin/amphotericin‐B). The U2OS bone osteosarcoma cell line was acquired from American Type Culture Collection (ATCC, Manassas, VA, USA) and cultured in Dulbecco's Modified Eagle's Medium (DMEM) supplemented with 10 % fetal bovine serum and 1 % penicillin. The cells were grown at 37 °C in a humidified atmosphere containing 5 % CO_2_. To gain access to OSC‐enriched cells, a full T75 flask of U2OS cells was treated with methotrexate (300 nM) for 4 days. The cells (labelled U2OS‐MTX cells) were then used immediately. U2OS‐MTX cells were characterised according to CD117 expression using flow cytometry as previously reported.^[37]^

**Antiproliferative studies: MTT assay**: Exponentially growing cells were seeded at a density of approximately 5×10^3^ cells per well in 96‐well flat‐bottomed microplates and allowed to attach for 24 h prior to addition of compounds. Various concentrations of the test compounds (0.0004–100 μM) were added and incubated for 72 h at 37 °C (total volume 200 μL). Stock solutions of the compounds were prepared as 10 mM DMSO solutions and diluted using cell media. The final concentration of DMSO in each well was ≤1 %. After 72 h, 20 μL of MTT (4 mg mL^−1^ in PBS) was added to each well and the plates incubated for an additional 4 h at 37 °C. The media/MTT mixture was eliminated and DMSO (100 μL per well) was added to dissolve the formazan precipitates. The optical density was measured at 550 nm using a 96‐well multiscanner autoreader. Absorbance values were normalised to (DMSO‐containing) control wells and plotted as concentration of compound versus % cell viability. IC_50_ values were interpolated from the resulting dose dependent curves. The reported IC_50_ values are the average of three independent experiments (n=18).

**Tumoursphere formation and viability assay**: HMLER‐shEcad cells (5×10^3^) were plated in ultralow‐attachment 96‐well plates (Corning) and incubated in MEGM supplemented with B27 (Invitrogen), 20 ng mL^−1^ EGF and 4 μg mL^−1^ heparin (Sigma) for 5 days. Studies were also conducted in the presence of **1**, **2**, cisplatin and salinomycin (0–133 μM). Mammospheres treated with **1**, **2**, cisplatin and salinomycin (at their respective IC_20_ values, 5 days) were counted and imaged using an inverted microscope. The viability of the mammospheres was determined by addition of a resazurin‐based reagent, TOX8 (Sigma). After incubation for 16 h, the fluorescence of the solutions was read at 590 nm (λ_ex_=560 nm). Viable mammospheres reduce the amount of the oxidised TOX8 form (blue) and concurrently increase the amount of the fluorescent TOX8 intermediate (red), indicating the degree of mammosphere cytotoxicity caused by the test compound. Fluorescence values were normalised to DMSO‐containing controls and plotted as concentration of test compound versus % mammospheres viability. IC_50_ values were interpolated from the resulting dose dependent curves. The reported IC_50_ values are the average of three independent experiments, each consisting of two replicates per concentration level (n=6).

**Cellular uptake**: To measure the cellular uptake of **1** about 1 million HMLER‐shEcad cells were treated with **1** (0.25 μM) at 37 °C for 24 h. After incubation, the media was removed, the cells were washed with PBS (2 mL×3) and harvested. The number of cells was counted at this stage, using a haemocytometer. This mitigates any cell death induced by **1** at the administered concentration and experimental cell loss. The cellular pellet was dissolved in 65 % HNO_3_ (250 μL) overnight. A cellular pellet of HMLER‐shEcad cells treated with **1** was also used to determine the copper content in the nuclear, cytoplasmic, and membrane fractions. The Thermo Scientific NE‐PER Nuclear and Cytoplasmic Extraction Kit was used to extract and separate the nuclear, cytoplasmic, and membrane fractions. The fractions were dissolved in 65 % HNO_3_ (250 μL final volume) overnight. All samples were diluted 17‐fold with water and analysed using inductively coupled plasma mass spectrometry (ICP‐MS, Thermo Scientific ICAP‐Qc quadrupole ICP mass spectrometer). Copper levels are expressed as mass of Cu (ng) per million cells. Results are presented as the mean of four determinations for each data point.

**Intracellular ROS assay**: HMLER‐shEcad cells (5×10^3^) were seeded in each well of a 96‐well plate. After incubating the cells overnight, they were treated with **1** (IC_50_ value ×2, 0.5–24 h) and incubated with 6‐carboxy‐2′,7′‐dichlorodihydrofluorescein diacetate (20 μM) for 90 min. The intracellular ROS level was determined by measuring the fluorescence of the solutions in each well at 529 nm (*λ*_ex_=504 nm).

**DNA cleavage studies**: Plasmid DNA (pUC19) was purchased from Invitrogen. The DNA cleavage activity of **1** was determined by monitoring the conversion of supercoiled plasmid DNA (form I) to smaller fragments of DNA in Tris‐HCl buffer (5 mM, pH 7.4), using agarose‐gel electrophoresis. To probe the effect of compound concentration on cleavage, solutions containing DNA (100 ng) and **1** (0–20 μM) without/with 10 equivalents of ascorbic acid, with a total reaction volume of 20 μL, were incubated at 37 °C for 24 h. To determine the oxidative cleavage mechanism, solutions containing DNA (100 ng), **1** (10 μM), 10 equivalents of ascorbic acid, and various radical scavenges (10 mM or 40 mM of ^t^BuOH, DMSO, KI, and NaN_3_), with a total reaction volume of 20 μL, were incubated at 37 °C for 24 h. Reactions were also conducted with DNA (100 ng), **1** (10 μM), 10 equivalents of ascorbic acid, and DAPI (50 μM), methyl green (50 μM), or thiazole orange (10 μM). After incubation, loading buffer (5 μL, containing 0.25 % bromophenol blue, 0.25 % xylene cyanol, and 60 % glycerol) was added and reaction mixtures were immediately loaded onto a 1 % agarose gel containing ethidium bromide (1.0 mg mL^−1^). The DNA fragments were separated by applying 60 V for 2 h in Tris‐acetate EDTA (TAE) buffer. The DNA bands were analyzed under UV light using a Bio‐Rad ChemiDoc Imaging System.

**Immunoblotting analysis**: HMLER‐shEcad cells (5×10^6^) were incubated with **1** (0.03–0.12 μM for 24 or 48 h) at 37 °C. HMLER‐shEcad cells were harvested and isolated as pellets. SDS‐PAGE loading buffer (64 mM Tris‐HCl (pH 6.8)/9.6 % glycerol/ 2 %SDS/5 % β‐mercaptoethanol/ 0.01 % bromophenol blue) was added to the pellets, and this was incubated at 95 °C for 10 min. Cell lysates were resolved by 4–20 % sodium dodecylsulphate polyacylamide gel electrophoresis (SDS‐PAGE; 200 V for 25 min) followed by electro transfer to polyvinylidene difluoride membrane, PVDF (350 mA for 1 h). Membranes were blocked in 5 % (w/v) non‐fat milk in PBST (PBS/0.1 % Tween 20) and incubated with the appropriate primary antibodies (Cell Signalling Technology). After incubation with horseradish peroxidase‐conjugated secondary antibodies (Cell Signalling Technology), immune complexes were detected with the ECL detection reagent (BioRad) and analysed using a chemiluminescence imager (Bio‐Rad ChemiDoc Imaging System).

**Annexin V‐propidium iodide assay**: HMLER‐shEcad cells were incubated with and without **1** (IC_50_ value ×2 for 48 h) and cisplatin (25 μM for 48 h) at 37 °C. Cells were harvested from adherent cultures by trypsinisation. The FITC Annexin V/Dead Cell Apoptosis Kit was used. The manufacture's (Thermo Fisher Scientific) protocol was followed to carry out this experiment. Briefly, untreated and treated cells (1×10^6^) were suspended in 1× Annexin binding buffer (100 μL) (10 mM HEPES, 140 mM NaCl, 2.5 mM CaCl_2_, pH 7.4), then 5 μL FITC Annexin V and 1 μL PI (100 μg/mL) were added to each sample and incubated at room temperature for 15 min. After which more 1× Annexin binding buffer (400 μL) was added while gently mixing. The cells were analysed using a FACSCanto II flow cytometer (BD Biosciences) (10,000 events per sample were acquired) at the University of Leicester FACS Facility. The FL1 channel was used to assess Annexin V binding and the FL2 channel was used to assess PI uptake. Cell populations were analysed using the FlowJo software (Tree Star).

## Conflict of interest

The authors declare no conflict of interest.

1

## Supporting information

As a service to our authors and readers, this journal provides supporting information supplied by the authors. Such materials are peer reviewed and may be re‐organized for online delivery, but are not copy‐edited or typeset. Technical support issues arising from supporting information (other than missing files) should be addressed to the authors.

Supporting Information

## Data Availability

The data that support the findings of this study are available from the corresponding author upon reasonable request.
