# Supplementary material for: The Bulk Breast Cancer Cell and Breast Cancer Stem Cell Activity of Binuclear Copper(II)‐Phenanthroline Complexes
Source: Chemistry. 2023 Jul 13;29(45):e202301188. doi: 10.1002/chem.202301188 (PMC10947161; doi:10.1002/chem.202301188)
Supplement: Supplementary file 1 — Supporting Information [file CHEM-29-0-s001.pdf]

Chemistry–A European Journal

Supporting Information

The Bulk Breast Cancer Cell and Breast Cancer Stem Cell Activity of Binuclear Copper(II)-Phenanthroline Complexes

Priscilla B. Osei, Joshua Northcote-Smith, Jiaxin Fang, Kuldip Singh, Fabrizio Ortu,* and Kogularamanan Suntharalingam*

<u>**Table of Content**</u>

Figure S1. (Top) Theoretical isotope model for [**1**-Cl]$^+$ ($C_{28}H_{24}Cl_3Cu_2N_4$) and (bottom) the experimentally determined high-resolution ESI-TOF mass spectrum for complex **1**.

Figure S2. (Top) Theoretical isotope model for [**2**-OH]$^+$ ($C_{52}H_{40}Cl_3Cu_2N_4$) and (bottom) the experimentally determined high-resolution ESI-TOF mass spectrum for complex **2**.

Figure S3. ATR-FTIR spectra of (A) **1** and (B) **2** in the solid form.

Table S1. Selected crystallographic data for complexes **1** and **2**.

Table S2. Selected bond lengths (Å) and angles (°) for complex **1**.

Table S3. Selected bond lengths (Å) and angles (°) for complex **2**.

Figure S4. UV-Vis spectrum of **1** (50 μM) in H_2O:DMSO (200:1) over the course of 24 h at 37 °C.

Figure S5. UV-Vis spectrum of **1** (50 μM) in PBS:DMSO (200:1) over the course of 24 h at 37 °C.

Figure S6. UV-Vis spectrum of **2** (50 μM) in H_2O:DMSO (200:1) over the course of 24 h at 37 °C.

Figure S7. UV-Vis spectrum of **2** (50 μM) in PBS:DMSO (200:1) over the course of 24 h at 37 °C.

Figure S8. UV-Vis spectrum of **1** (50 μM) in the presence of ascorbic acid (500 μM) in PBS:DMSO (200:1) over the course of 24 h at 37 °C.

Figure S9. UV-Vis spectrum of **2** (50 μM) in the presence of ascorbic acid (500 μM) in PBS:DMSO (200:1) over the course of 24 h at 37 °C.

Figure S10. UV-Vis spectrum of **1** (25 μM) in the presence of glutathione (250 μM) in PBS:DMSO (200:1) over the course of 24 h at 37 °C.

Figure S11. UV-Vis spectrum of **2** (25 μM) in the presence of glutathione (250 μM) in PBS:DMSO (200:1) over the course of 24 h at 37 °C.

Figure S12. UV-Vis spectrum of **1** (25 μM) in the presence of NADH (250 μM) in PBS:DMSO (200:1) over the course of 24 h at 37 °C.

Figure S13. UV-Vis spectrum of **2** (25 μM) in the presence of NADH (250 μM) in PBS:DMSO (200:1) over the course of 24 h at 37 °C.

Figure S14. UV-Vis spectrum of **2** (50 μM) in the presence of ascorbic acid (500 μM) and bathocuproine disulfonate, BCS (100 μM) in PBS:DMSO (200:1) over the course of 24 h at 37 °C.

Figure S15. High resolution ESI mass spectrum (positive mode) of **2** (500 μM) in H_2O:DMSO (10:1) in the presence of ascorbic acid (5 mM) after 24 h incubation at 37 °C.

Figure S16. High resolution ESI mass spectrum (positive mode) of **1** (500 μM) in H_2O:DMSO (10:1) in the presence of glutathione (5 mM) after 24 h incubation at 37 °C.

Figure S17. High resolution ESI mass spectrum (positive mode) of **2** (500 μM) in H_2O:DMSO (10:1) in the presence of glutathione (5 mM) after 24 h incubation at 37 °C.

Figure S18. High resolution ESI mass spectrum (positive mode) of **1** (500 μM) in H_2O:DMSO (10:1) in the presence of NADH (5 mM) after 24 h incubation at 37 °C.

Figure S19. High resolution ESI mass spectrum (positive mode) of **2** (500 μM) in H_2O:DMSO (10:1) in the presence of NADH (5 mM) after 24 h incubation at 37 °C.

Figure S20. Representative dose-response curves for the treatment of HMLER and HMLER-shEcad cells with **1** after 72 h incubation.

Figure S21. Representative dose-response curves for the treatment of HMLER and HMLER-shEcad cells with **2** after 72 h incubation.

Figure S22. Representative dose-response curves for the treatment of MCF10A cells with **1** or **2** after 72 h incubation.

Figure S23. Representative dose-response curves for the treatment of HMLER and HMLER-shEcad cells with Cu(1,10-phenanthroline)Cl_2 after 72 h incubation.

Figure S24. Representative dose-response curves for the treatment of HMLER and HMLER-shEcad cells with Cu(4,7-diphenyl-1,10-phenanthroline)Cl_2 after 72 h incubation.

Table S4. IC_{50} values of Cu(1,10-phenanthroline)Cl_2, and Cu(4,7-diphenyl-1,10-phenanthroline)Cl_2 against HMLER and HMLER-shEcad cells. [a] Determined after 72 h incubation (mean of three independent experiments ± SD).

Figure S25. Representative dose-response curves for the treatment of U2OS and U2OS-MTX cells with **1** after 72 h incubation.

Figure S26. Representative dose-response curves for the treatment of U2OS and U2OS-MTX cells with **2** after 72 h incubation.

Table S5. IC_{50} values of **1** and **2** against U2OS and U2OS-MTX cells. [a] Determined after 72 h incubation (mean of three independent experiments ± SD).

Figure S27. Representative bright-field images (× 10) of HMLER-shEcad spheroids in the absence and presence of salinomycin at its IC_{20} value (5 days incubation).

Figure S28. FITC Annexin V-propidium iodide binding assay plots of untreated HMLER-shEcad cells and HMLER-shEcad cells treated with **1** (0.25 μM for 24 h).

Figure S29. Concentration-dependent DNA cleavage by **1** after a 24 h incubation period. Lane 1: DNA only, Lane 2-3: DNA + 10 and 20 μM of **1**.

Figure S30. Inhibition of **1**-mediated DNA cleavage by DNA minor and major groove binders and an intercalator after 24 h incubation, Lane 1: DNA only, Lane 2: DNA + **1** (10 μM) with 10 equivalents of ascorbic acid, Lane 3: DNA + **1** (10 μM) with 10 equivalents of ascorbic acid + DAPI (50 μM), Lane 4: DNA + **1** (10 μM) with 10 equivalents of ascorbic acid + methyl green (50 μM), Lane 5: DNA + **1** (10 μM) with 10 equivalents of ascorbic acid + TO (10 μM).

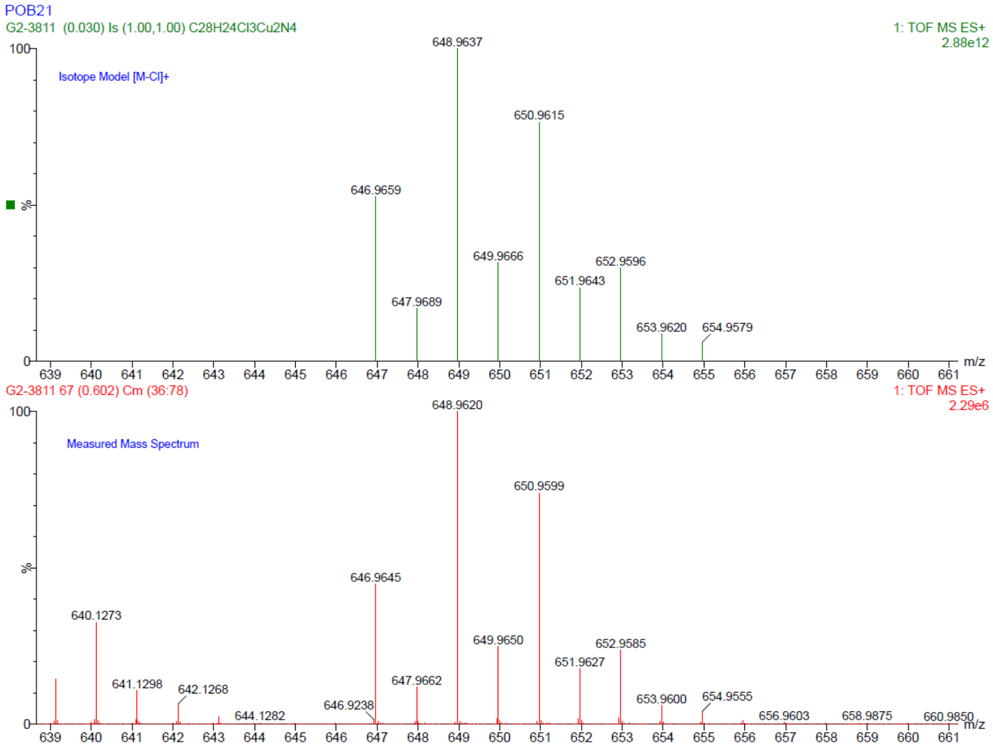

Figure S1. (Top) Theoretical isotope model for [**1**-Cl]$^{+}$ ($C_{28}H_{24}Cl_3Cu_2N_4$) and (bottom) the experimentally determined high-resolution ESI-TOF mass spectrum for complex **1**.

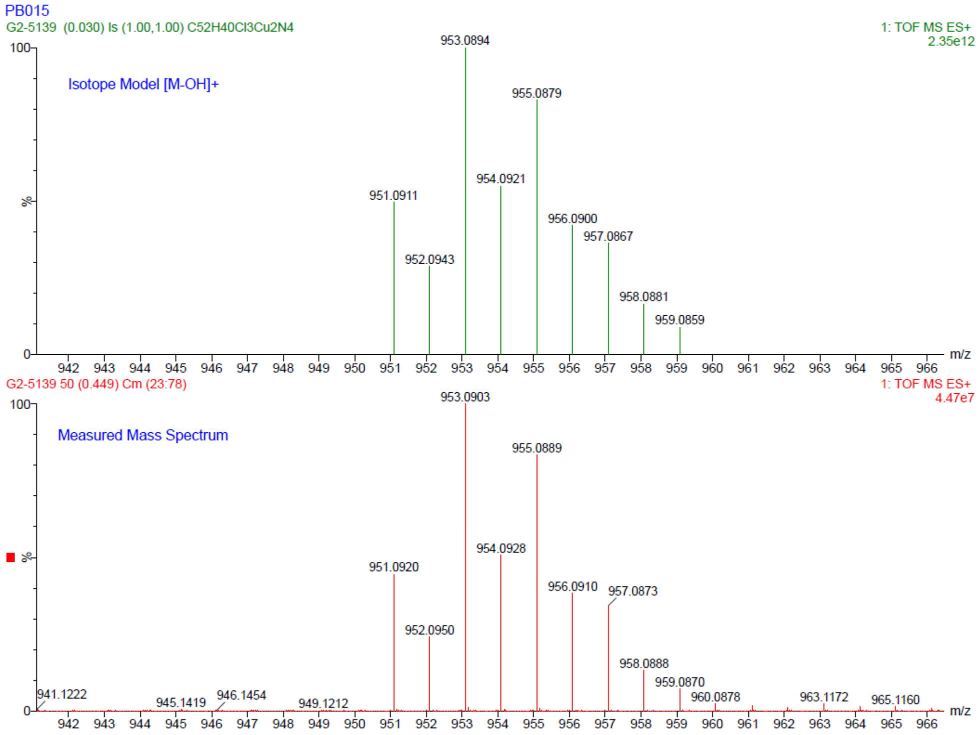

Figure S2. (Top) Theoretical isotope model for [**2**-OH]$^{+}$ ($C_{52}H_{40}Cl_3Cu_2N_4$) and (bottom) the experimentally determined high-resolution ESI-TOF mass spectrum for complex **2**.

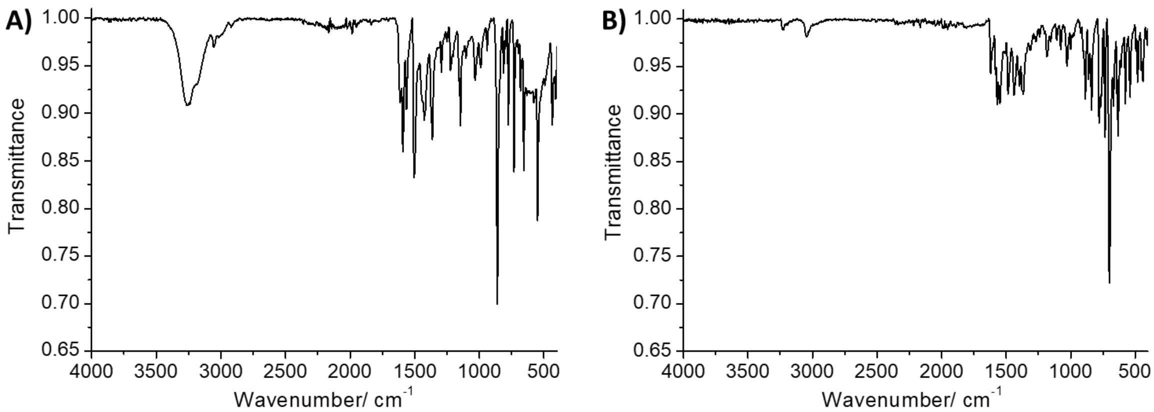

Figure S3. ATR-FTIR spectra of (A) **1** and (B) **2** in the solid form.

Table S1. Selected crystallographic data for complexes **1** and **2**.

**Metal complex**	**1**	**2**
CCDC No.	2250995	2250994
formula	$C_{28}H_{24}Cl_4Cu_2N_4$	$C_{52}H_{41}Cl_3Cu_2N_4O\cdot 1.5Et_2O$
w	685.39	1082.49
Crystal system	triclinic	monoclinic
Space group	*P-1*	$P2_1/n$
a, Å	7.2891(3)	7.5567(3)
b, Å	9.3645(4)	41.2370(14)
c, Å	10.9293(5)	16.6384(6)
α, deg.	109.527(2)	90
β, deg.	104.499(2)	91.9610(10)
γ, deg.	93.426(2)	90
V, Å^3	672.26(5)	5181.7(3)
Z	1	4
ρ_{calc}g/cm^3	1.693	1.388
2θ / deg.	8.962 to 144.636	5.73 to 144.512
Reflections collected	15694	41220
Independent reflections	2586	10212
Goodness-of-fit on F^2	1.059	1.056
R_1, wR_2 [$I\geq 2\sigma(I)$]	0.0333, 0.0914	0.0294, 0.0757
R_1, wR_2 [all data]	0.0343, 0.0925	0.0311, 0.0767
Largest diff. peak/hole / e Å^{-3}	0.65/-0.40	0.29/-0.34

Table S2. Selected bond lengths (Å) and angles (°) for complex **1**.

Cu(1)-Cl(1)^{i}	2.3471(5)	Cu(1)-N(1)	2.266(2)
Cu(1)-Cl(1)	2.3333(5)	Cu(1)-N(2)	2.017(2)
Cu(1)-Cl(2)	2.2580(5)		
Cl(1)-Cu(1)-Cl(1)^{i}	83.297(19)	N(1)-Cu(1)-Cl(1)^{i}	97.12(4)
Cl(2)-Cu(1)-Cl(1)^{i}	156.41(2)	N(2)-Cu(1)-Cl(1)^{i}	90.34(5)
Cl(2)-Cu(1)-Cl(1)	90.56(2)	N(2)-Cu(1)-Cl(1)	169.32(5)
Cl(2)-Cu(1)-N(1)	106.36(4)	N(2)-Cu(1)-Cl(2)	91.91(5)
N(1)-Cu(1)-Cl(1)	110.24(5)	N(2)-Cu(1)-N(1)	78.98(7)

Table S3. Selected bond lengths (Å) and angles (°) for complex **2**.

Cu(1)-Cl(1)	2.3093(4)	Cu(2)-Cl(2)	2.2986(5)
Cu(1)-Cl(3)	2.4373(4)	Cu(2)-Cl(3)	2.3922(4)
Cu(1)-O(1)	1.9114(11)	Cu(2)-O(1)	1.9181(11)
Cu(1)-N(2)	2.2178(13)	Cu(2)-N(1)	2.0164(13)
Cu(1)-N(4)	1.9946(13)	Cu(2)-N(3)	2.2431(13)
Cl(1)-Cu(1)-Cl(3)	156.62(2)	N(2)-Cu(1)-Cl(1)	101.86(3)
O(1)-Cu(1)-Cl(1)	93.35(4)	N(2)-Cu(1)-Cl(3)	101.50(3)
O(1)-Cu(1)-Cl(3)	79.48(4)	N(4)-Cu(1)-Cl(1)	93.93(4)
O(1)-Cu(1)-N(2)	109.25(5)	N(4)-Cu(1)-Cl(3)	90.00(4)
O(1)-Cu(1)-N(4)	167.76(5)	N(4)-Cu(1)-N(2)	78.80(5)
Cl(2)-Cu(2)-Cl(3)	152.47(2)	N(1)-Cu(2)-Cl(2)	92.26(4)
O(1)-Cu(2)-Cl(2)	91.29(4)	N(1)-Cu(2)-Cl(3)	92.68(4)
O(1)-Cu(2)-Cl(3)	80.54(3)	N(1)-Cu(2)-N(3)	78.16(5)
O(1)-Cu(2)-N(1)	171.23(5)	N(3)-Cu(2)-Cl(2)	103.26(4)
O(1)-Cu(2)-N(3)	108.79(5)	N(3)-Cu(2)-Cl(3)	104.26(4)

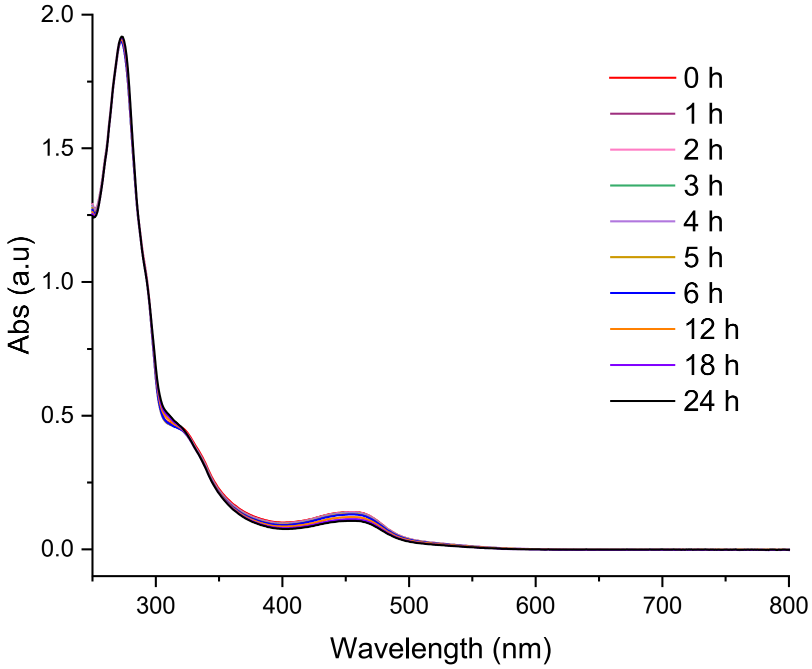

Figure S4. UV-Vis spectrum of **1** (50 μM) in H_2O:DMSO (200:1) over the course of 24 h at 37 ºC.

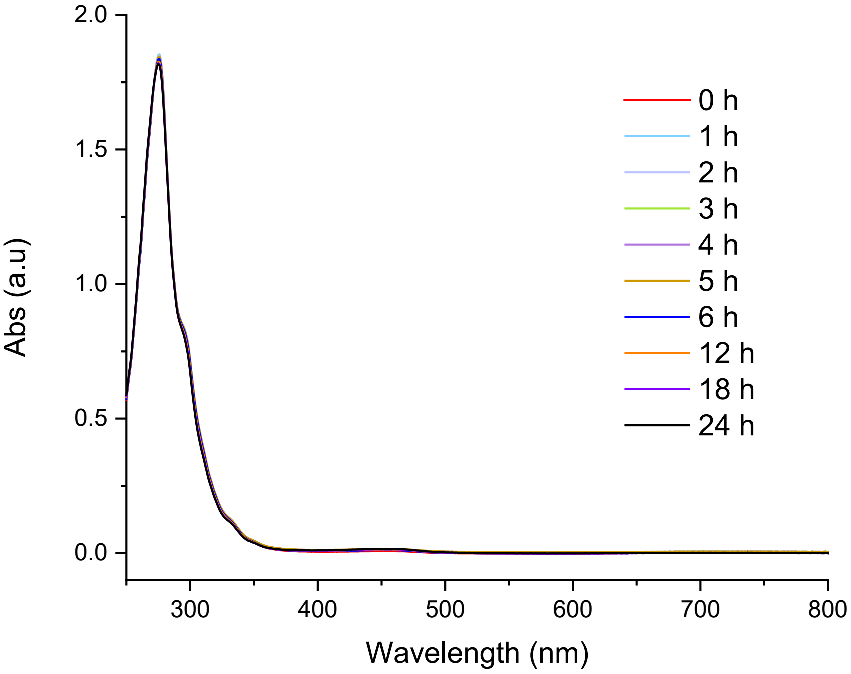

Figure S5. UV-Vis spectrum of **1** (50 μM) in PBS:DMSO (200:1) over the course of 24 h at 37 ºC.

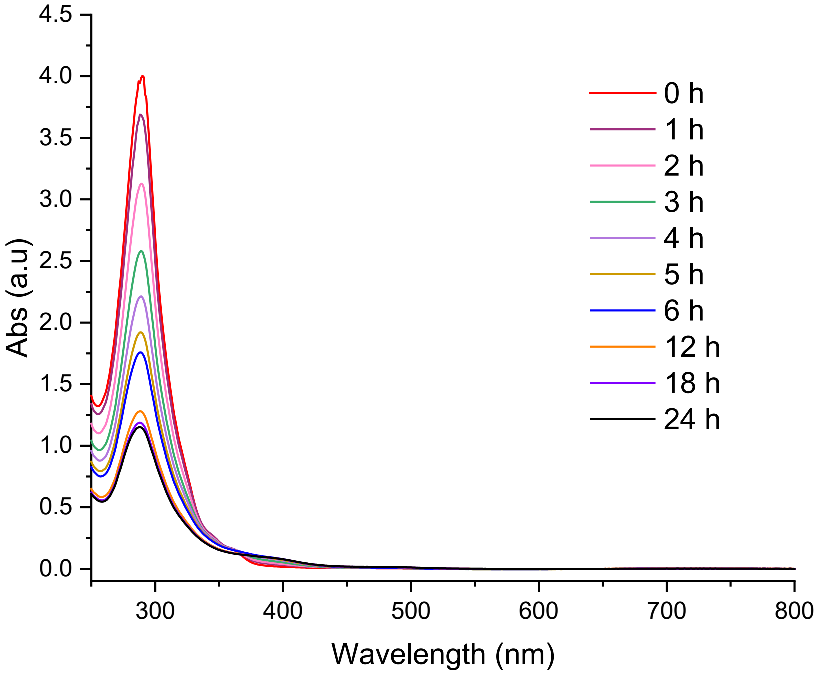

Figure S6. UV-Vis spectrum of **2** (50 μM) in H_2O:DMSO (200:1) over the course of 24 h at 37 ºC.

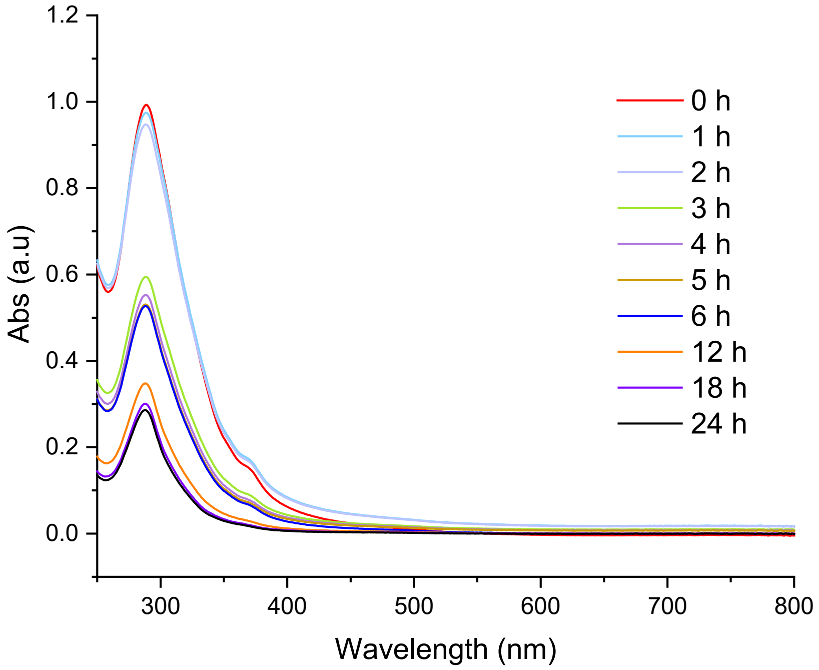

Figure S7. UV-Vis spectrum of **2** (50 μM) in PBS:DMSO (200:1) over the course of 24 h at 37 ºC.

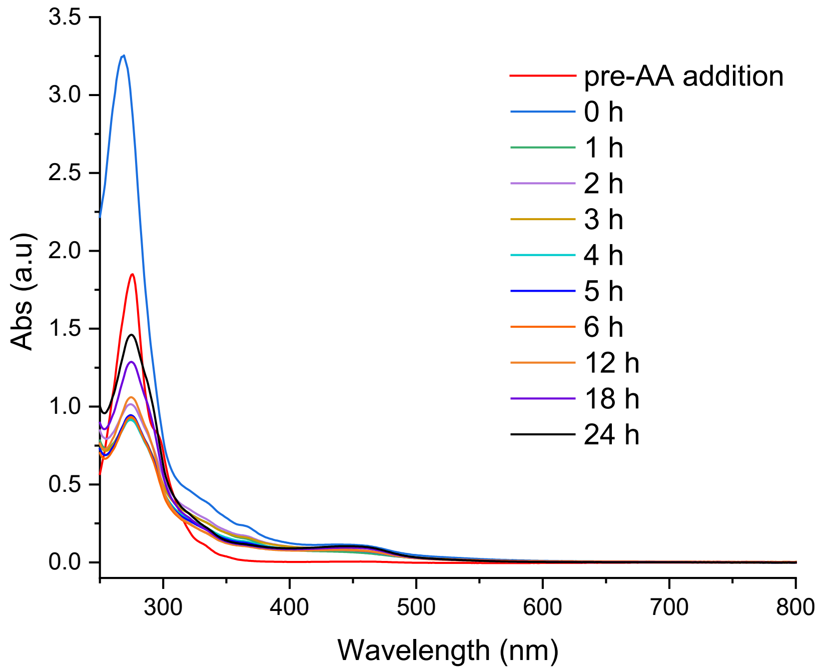

Figure S8. UV-Vis spectrum of **1** (50 µM) in the presence of ascorbic acid (500 µM) in PBS:DMSO (200:1) over the course of 24 h at 37 ºC.

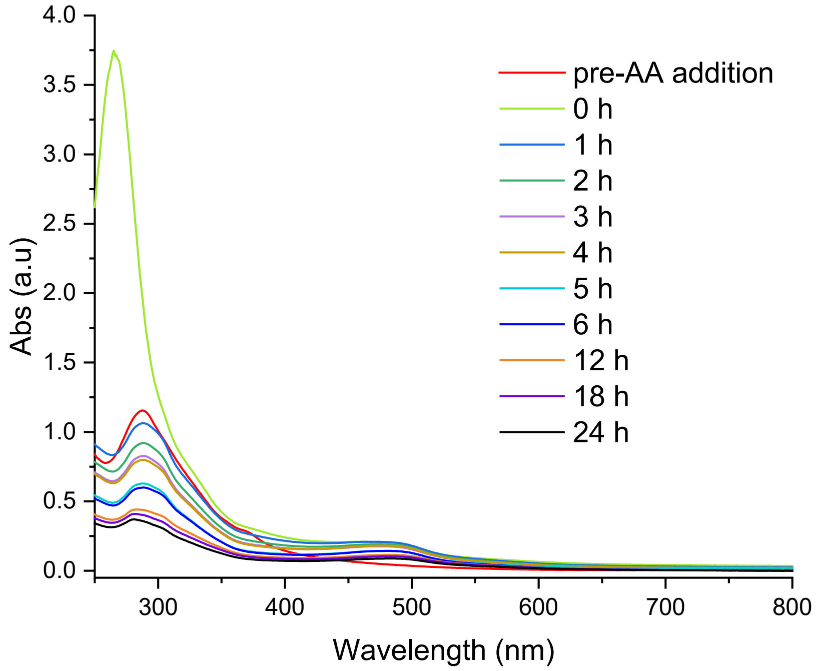

Figure S9. UV-Vis spectrum of **2** (50 µM) in the presence of ascorbic acid (500 µM) in PBS:DMSO (200:1) over the course of 24 h at 37 ºC.

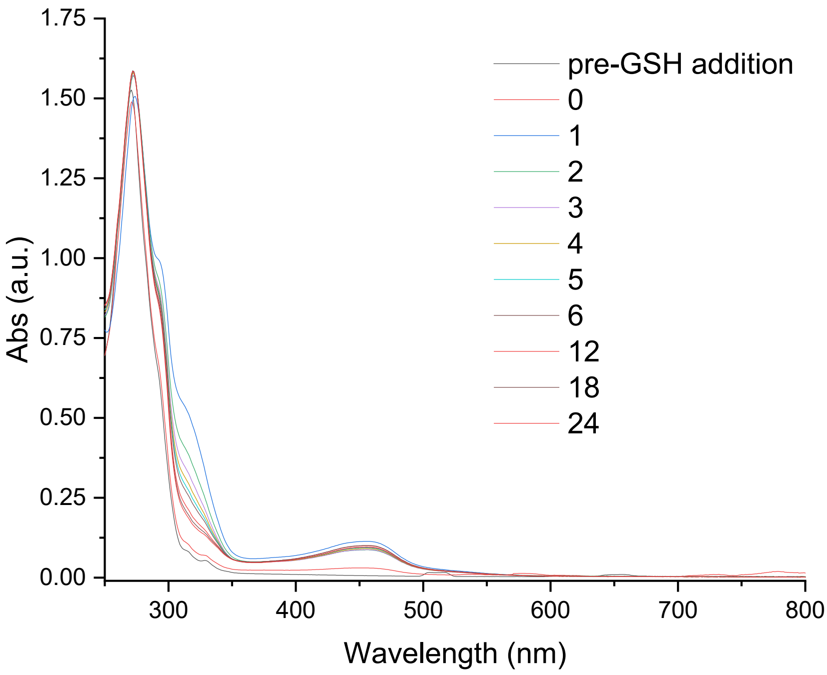

Figure S10. UV-Vis spectrum of **1** (25 μM) in the presence of glutathione (250 μM) in PBS:DMSO (200:1) over the course of 24 h at 37 ºC.

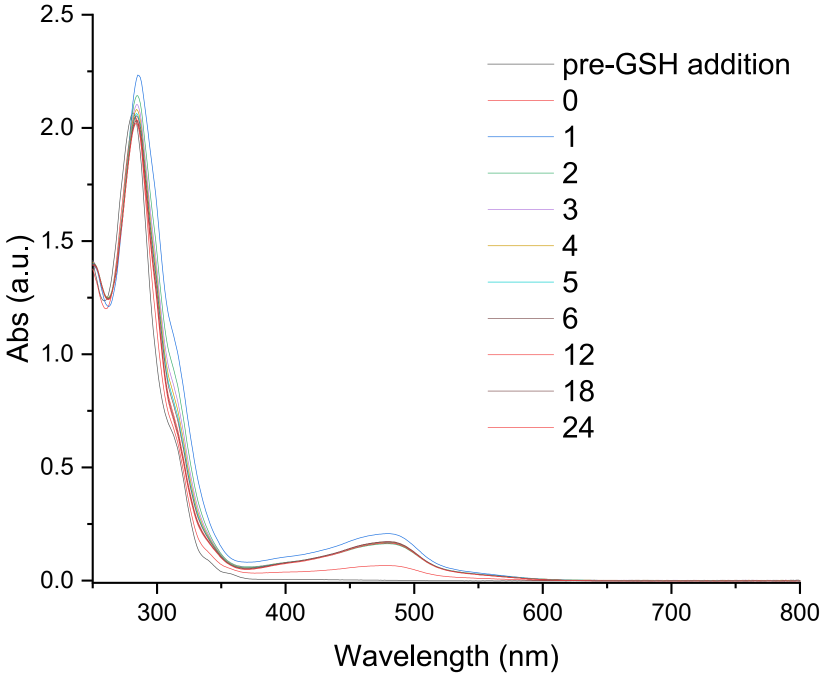

Figure S11. UV-Vis spectrum of **2** (25 μM) in the presence of glutathione (250 μM) in PBS:DMSO (200:1) over the course of 24 h at 37 ºC.

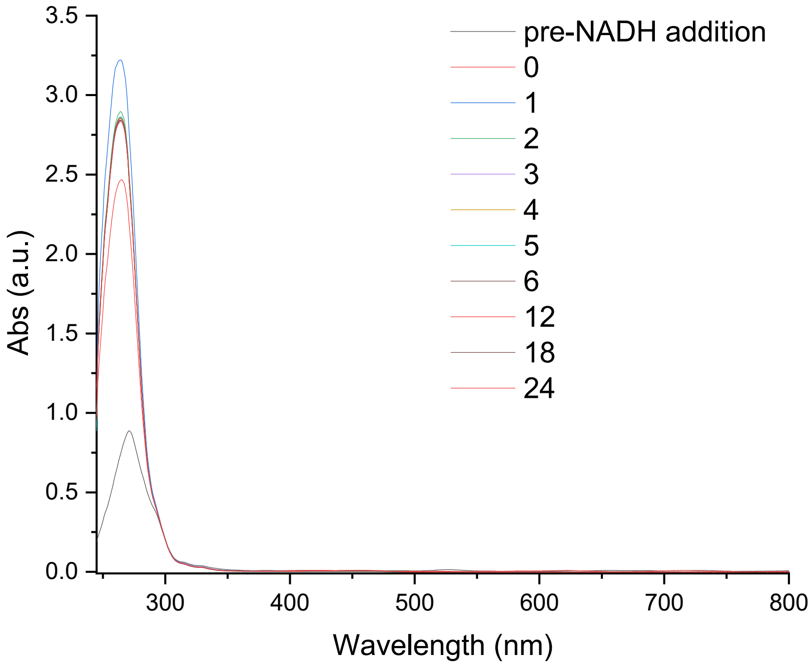

Figure S12. UV-Vis spectrum of **1** (25 μM) in the presence of NADH (250 μM) in PBS:DMSO (200:1) over the course of 24 h at 37 ºC.

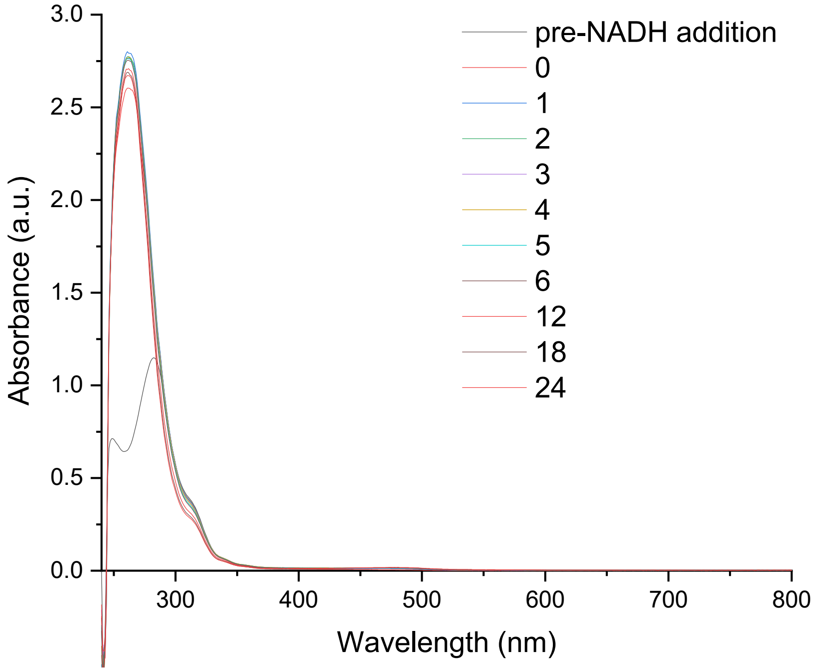

Figure S13. UV-Vis spectrum of **2** (25 μM) in the presence of NADH (250 μM) in PBS:DMSO (200:1) over the course of 24 h at 37 ºC.

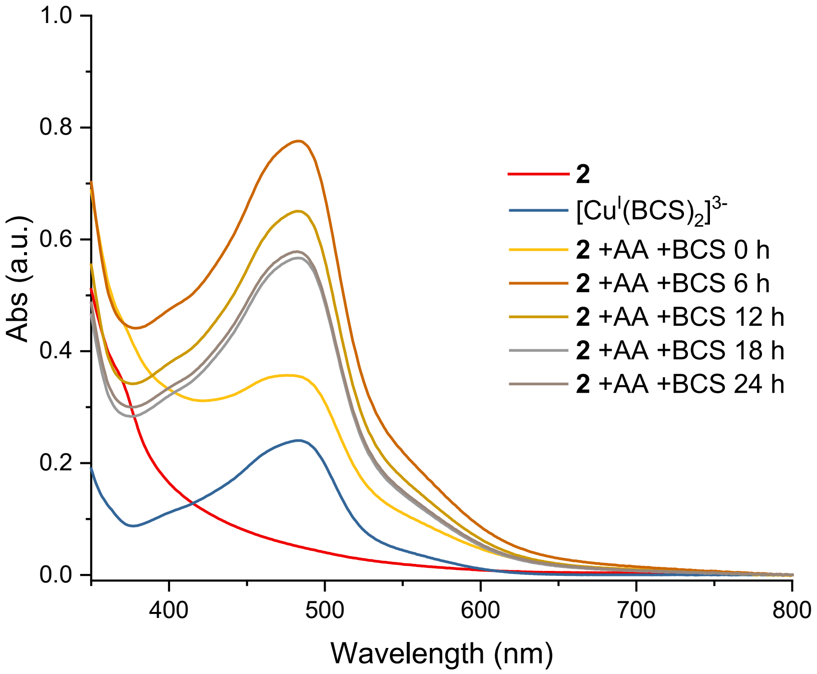

Figure S14. UV-Vis spectrum of **2** (50 μM) in the presence of ascorbic acid (500 μM) and bathocuproine disulfonate, BCS (100 μM) in PBS:DMSO (200:1) over the course of 24 h at 37 °C.

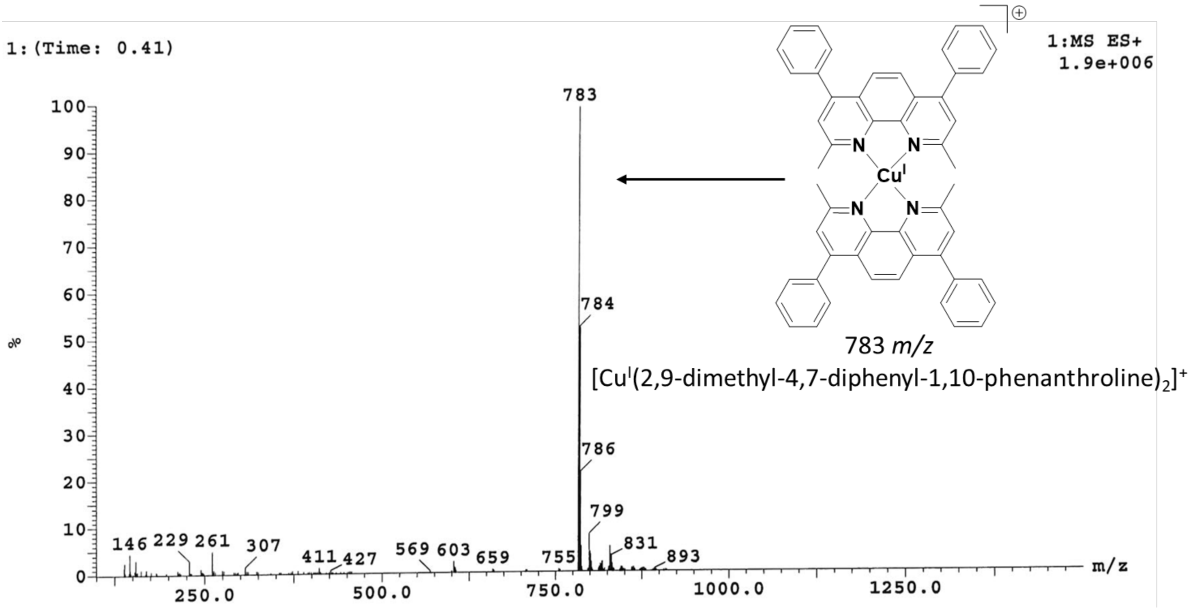

Figure S15. High resolution ESI mass spectrum (positive mode) of **2** (500 μM) in H_2O:DMSO (10:1) in the presence of ascorbic acid (5 mM) after 24 h incubation at 37 °C.

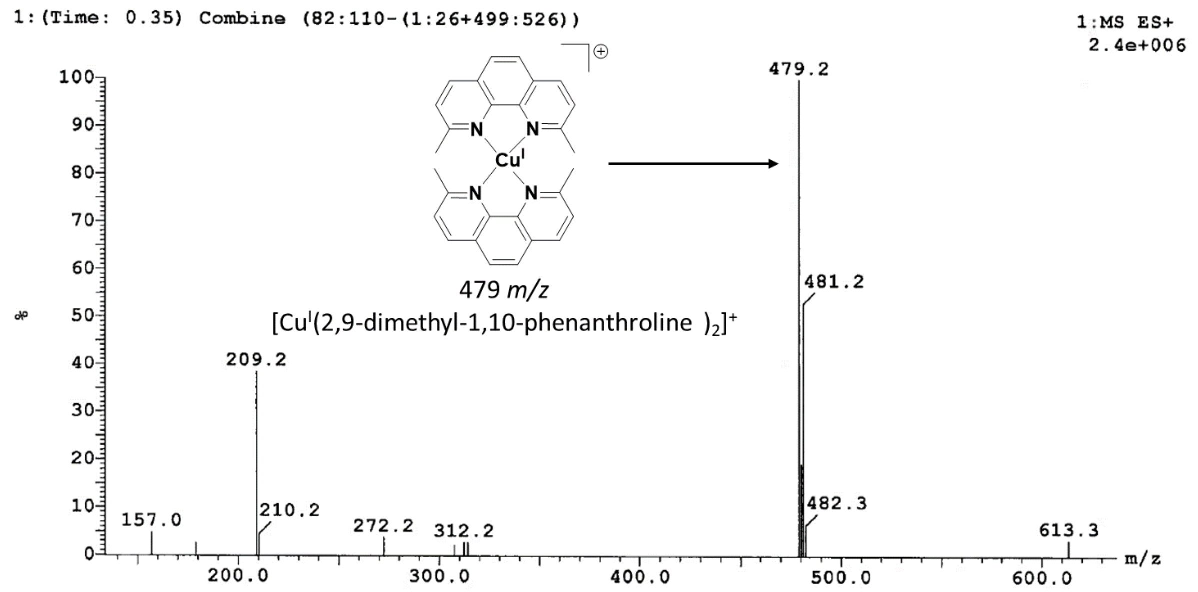

Figure S16. High resolution ESI mass spectrum (positive mode) of **1** (500 µM) in H_2O:DMSO (10:1) in the presence of glutathione (5 mM) after 24 h incubation at 37 ºC.

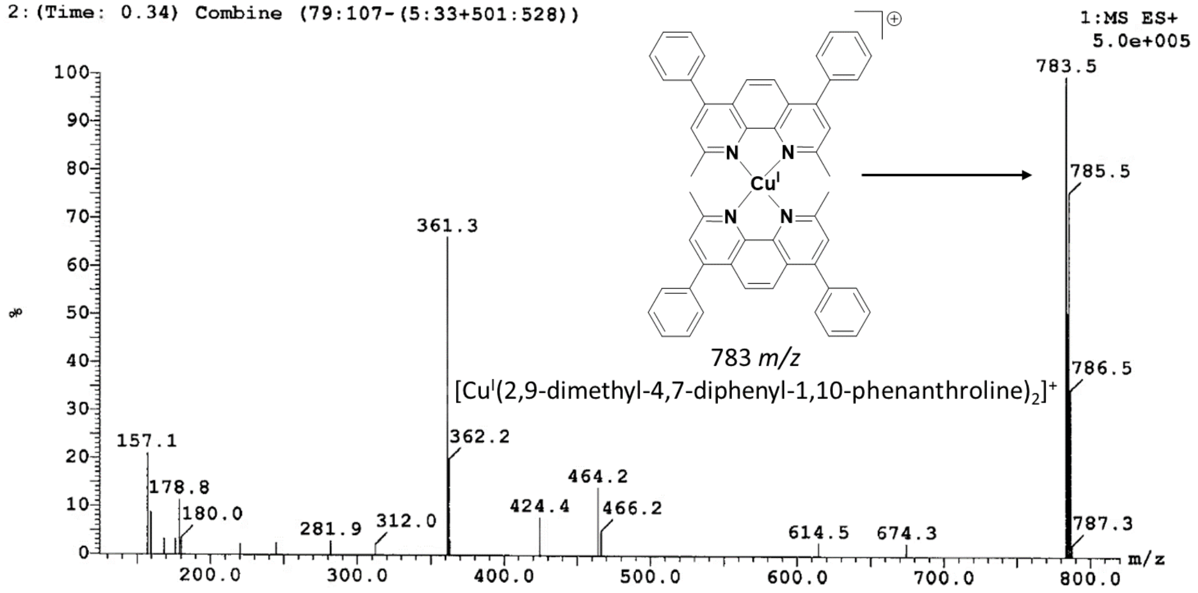

Figure S17. High resolution ESI mass spectrum (positive mode) of **2** (500 µM) in H_2O:DMSO (10:1) in the presence of glutathione (5 mM) after 24 h incubation at 37 ºC.

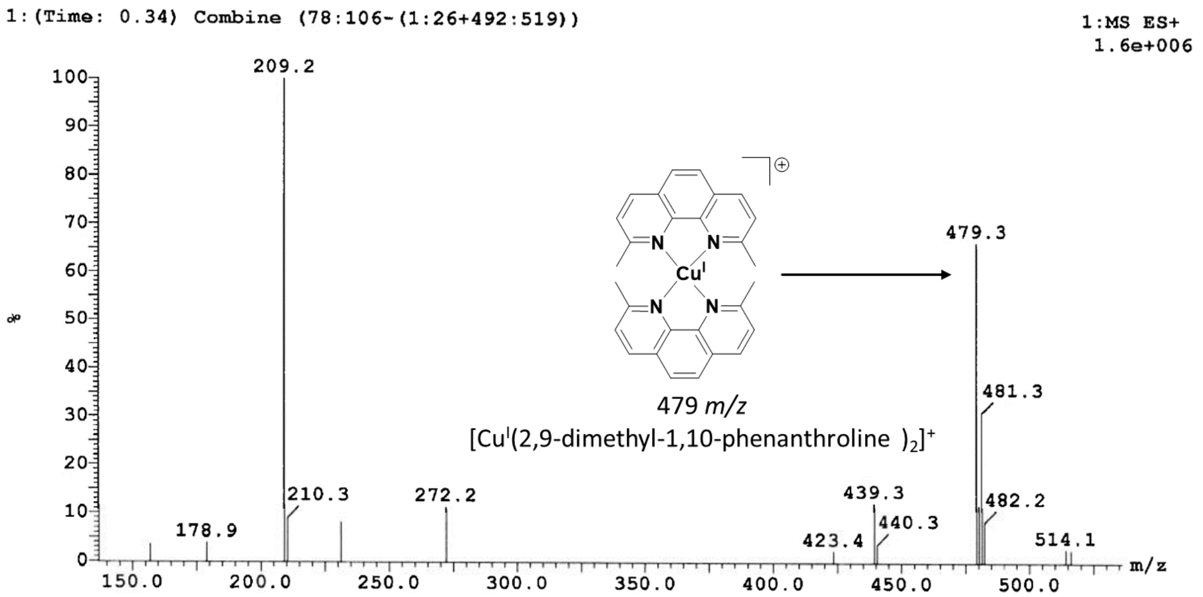

Figure S18. High resolution ESI mass spectrum (positive mode) of **1** (500 μM) in H_2O:DMSO (10:1) in the presence of NADH (5 mM) after 24 h incubation at 37 °C.

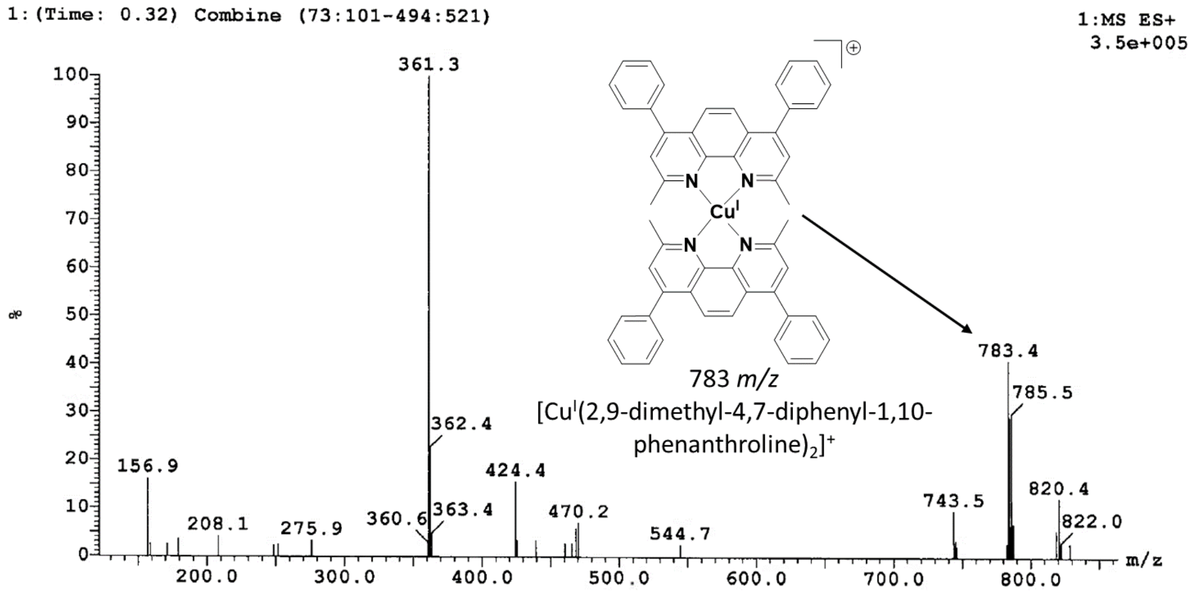

Figure S19. High resolution ESI mass spectrum (positive mode) of **2** (500 μM) in H_2O:DMSO (10:1) in the presence of NADH (5 mM) after 24 h incubation at 37 °C.

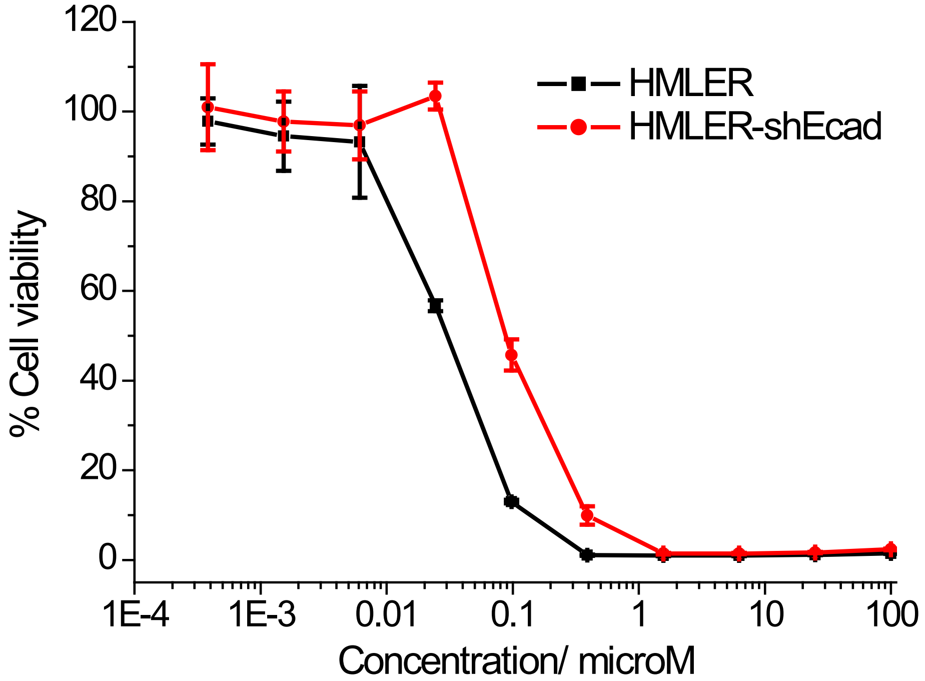

Figure S20. Representative dose-response curves for the treatment of HMLER and HMLER-shEcad cells with **1** after 72 h incubation.

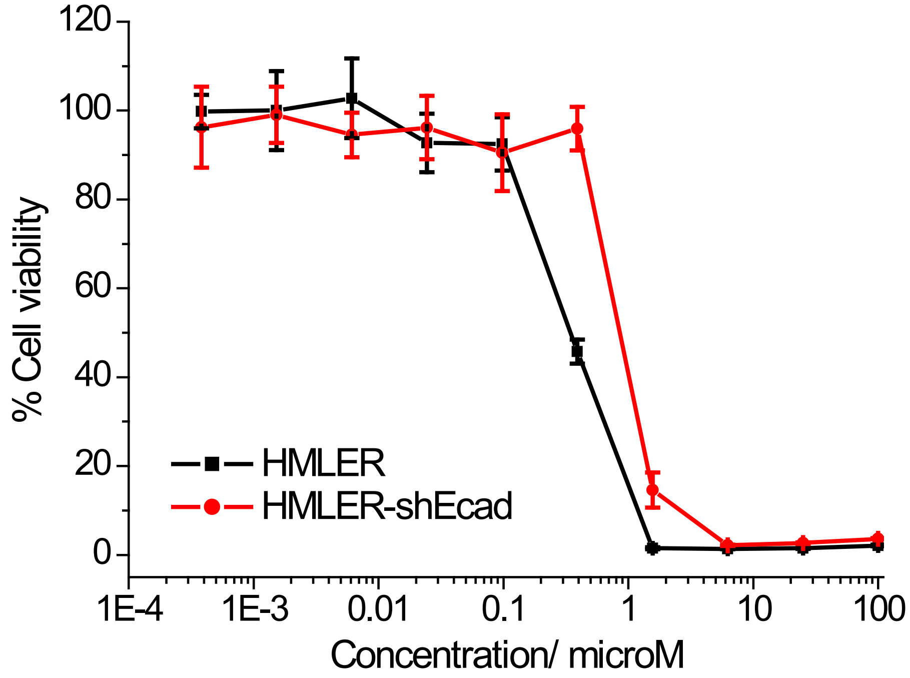

Figure S21. Representative dose-response curves for the treatment of HMLER and HMLER-shEcad cells with **2** after 72 h incubation.

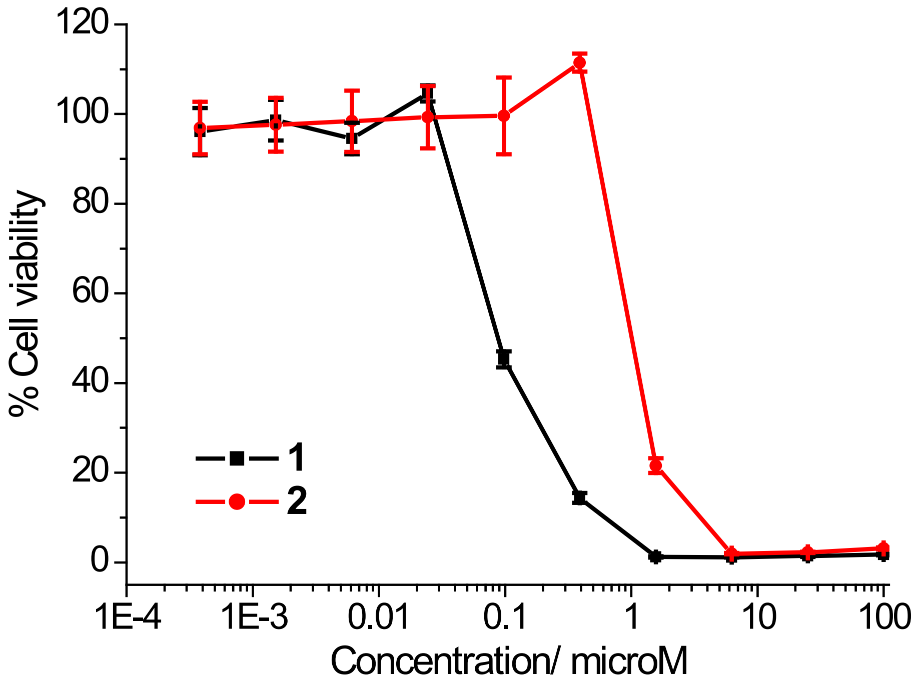

Figure S22. Representative dose-response curves for the treatment of MCF10A cells with **1** or **2** after 72 h incubation.

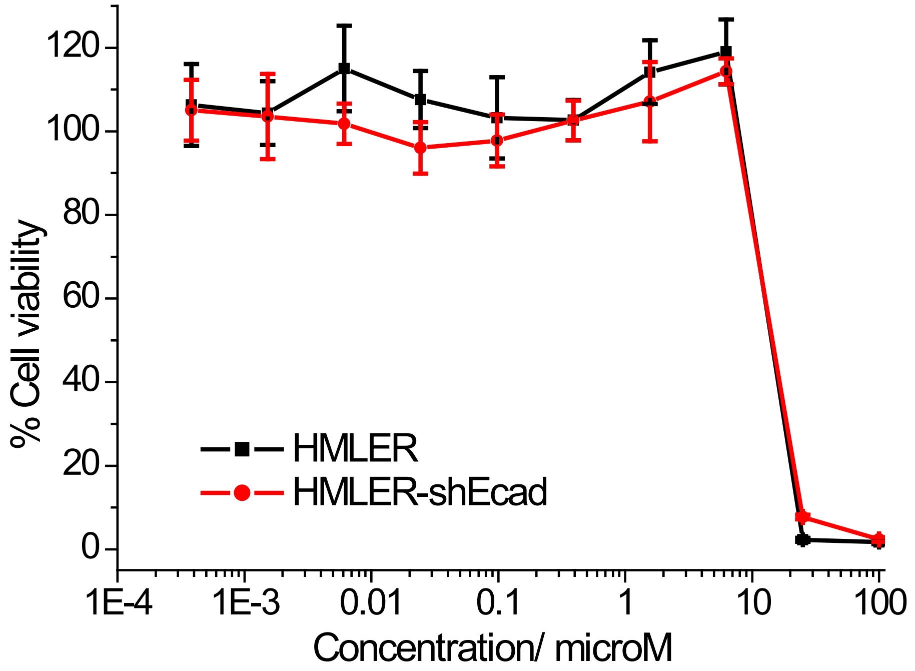

Figure S23. Representative dose-response curves for the treatment of HMLER and HMLER-shEcad cells with Cu(1,10-phenanthroline)Cl_2 after 72 h incubation.

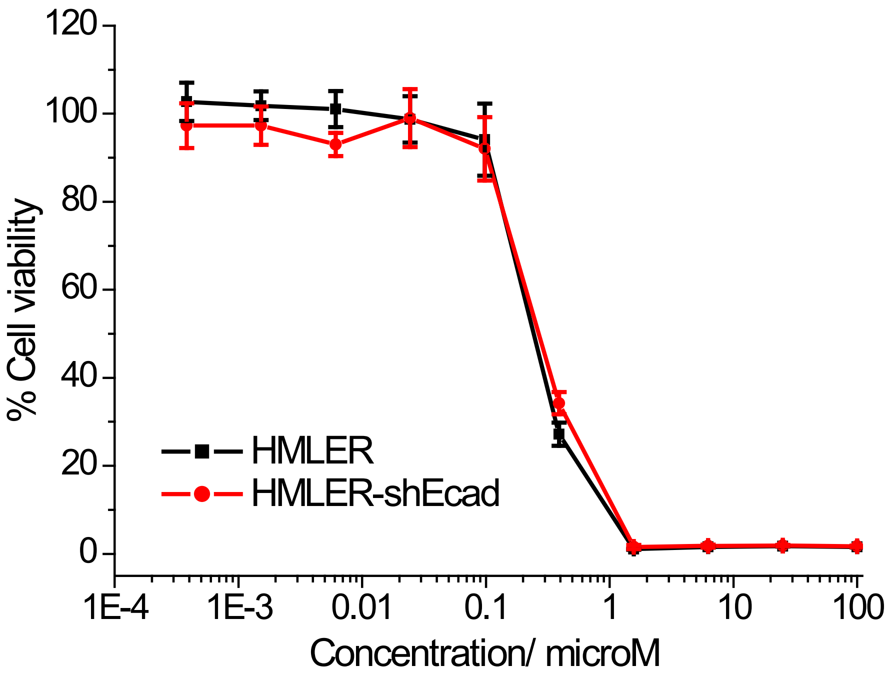

Figure S24. Representative dose-response curves for the treatment of HMLER and HMLER-shEcad cells with Cu(4,7-diphenyl-1,10-phenanthroline)Cl_2 after 72 h incubation.

Table S4. IC_{50} values of Cu(1,10-phenanthroline)Cl_2, and Cu(4,7-diphenyl-1,10-phenanthroline)Cl_2 against HMLER and HMLER-shEcad cells. [a] Determined after 72 h incubation (mean of three independent experiments ± SD).

Compound	HMLER [μM] [a]	HMLER-shEcad [μM] [a]
Cu(1,10-phenanthroline)Cl_2	14.25 ± 0.21	14.45 ± 0.21
Cu(4,7-diphenyl-1,10-phenanthroline)Cl_2	0.24 ± 0.02	0.27 ± 0.01

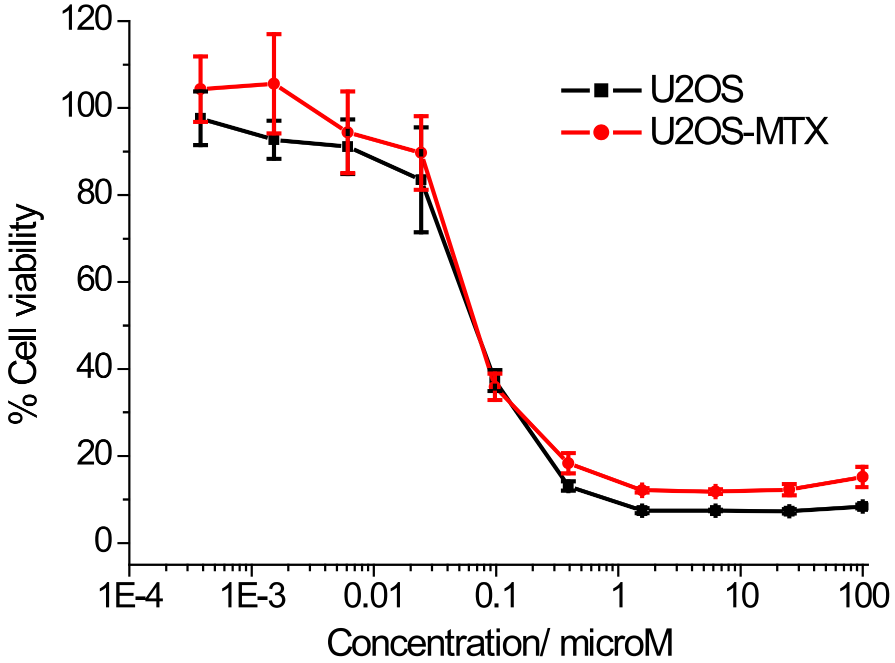

Figure S25. Representative dose-response curves for the treatment of U2OS and U2OS-MTX cells with **1** after 72 h incubation.

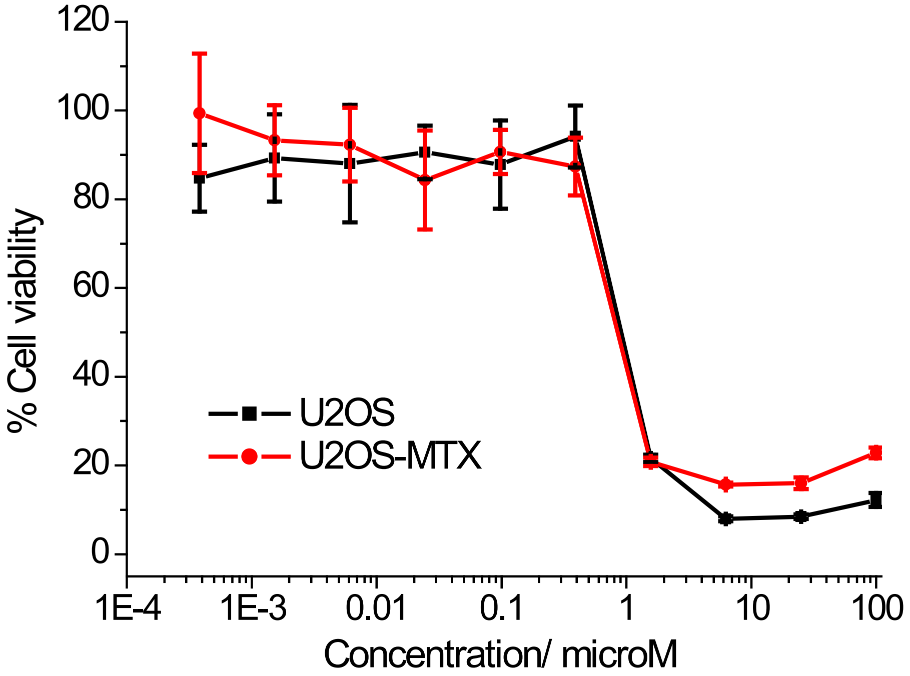

Figure S26. Representative dose-response curves for the treatment of U2OS and U2OS-MTX cells with **2** after 72 h incubation.

Table S5. IC_{50} values of **1** and **2** against U2OS and U2OS-MTX cells. [a] Determined after 72 h incubation (mean of three independent experiments ± SD).

Compound	U2OS [µM] [a]	U2OS-MTX [µM] [a]
**1**	0.07 ± 0.001	0.07 ± 0.01
**2**	0.91 ± 0.01	0.85 ± 0.02

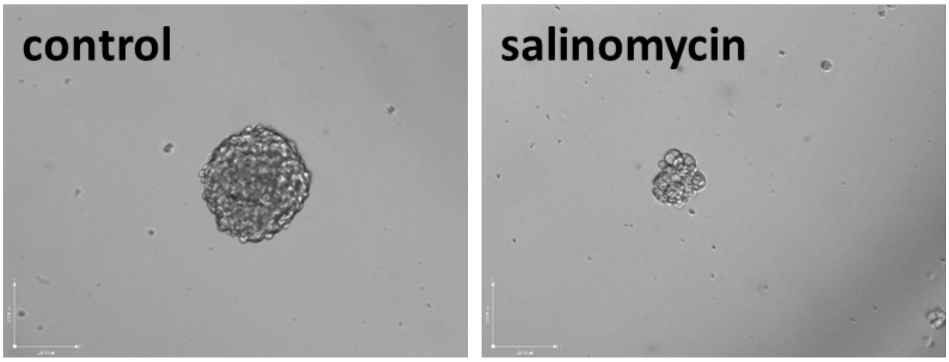

Figure S27. Representative bright-field images (× 10) of HMLER-shEcad spheroids in the absence and presence of salinomycin at its IC_{20} value (5 days incubation).

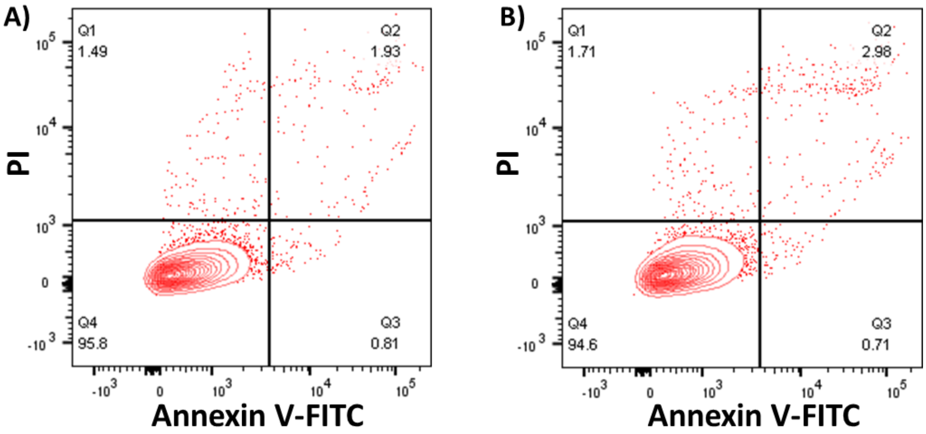

Figure S28. FITC Annexin V-propidium iodide binding assay plots of untreated HMLER-shEcad cells and HMLER-shEcad cells treated with **1** (0.25 µM for 24 h).

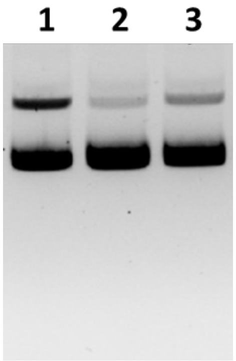

Figure S29. Concentration-dependent DNA cleavage by **1** after a 24 h incubation period. Lane 1: DNA only, Lane 2-3: DNA + 10 and 20 μM of **1**.

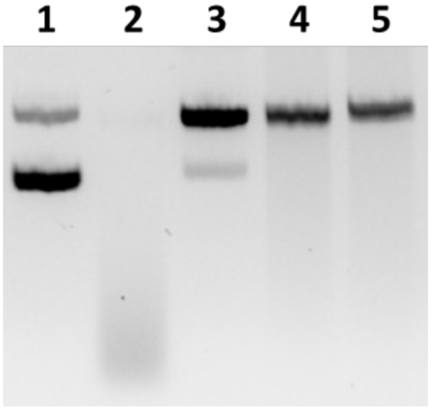

Figure S30. Inhibition of **1**-mediated DNA cleavage by DNA minor and major groove binders and an intercalator after 24 h incubation, Lane 1: DNA only, Lane 2: DNA + **1** (10 μM) with 10 equivalents of ascorbic acid, Lane 3: DNA + **1** (10 μM) with 10 equivalents of ascorbic acid + DAPI (50 μM), Lane 4: DNA + **1** (10 μM) with 10 equivalents of ascorbic acid + methyl green (50 μM), Lane 5: DNA + **1** (10 μM) with 10 equivalents of ascorbic acid + TO (10 μM).
